# Quantitative Proteomic Profiling of Marine Diatom *Skeletonema dohrnii* in Response to Temperature and Silicate Induced Environmental Stress

**DOI:** 10.3389/fmicb.2020.554832

**Published:** 2021-01-14

**Authors:** Satheeswaran Thangaraj, Satheesh Kumar Palanisamy, Guicheng Zhang, Jun Sun

**Affiliations:** ^1^College of Marine Science and Technology, China University of Geosciences, Wuhan, China; ^2^Department of Zoology, School of Natural Science, Ryan Institute, National University of Ireland, Galway, Ireland; ^3^Research Center for Indian Ocean Ecosystem, Tianjin University of Science and Technology, Tianjin, China; ^4^Tianjin Key Laboratory of Marine Resources and Chemistry, Tianjin University of Science and Technology, Tianjin, China

**Keywords:** global warming, photosynthesis, carbon metabolism, nutrient stratifications, iTRAQ-proteomics, climate change, biomarkers, ribosome biogenesis

## Abstract

Global warming is expected to reduce the nutrient concentration in the upper ocean and affect the physiology of marine diatoms, but the underlying molecular mechanisms controlling these physiological changes are currently unknown. To understand these mechanisms, here we investigated iTRAQ based proteomic profiling of diatom *Skeletonema dohrnii* in a multifactorial experimental with a combining change of temperature and silicate concentrations. In total, 3369 differently abundant proteins were detected in four different environmental conditions, and the function of all proteins was identified using Gene Ontology and KEGG pathway analysis. For discriminating the proteome variation among samples, multivariate statistical analysis (PCA, PLS-DA) was performed by comparing the protein ratio differences. Further, performing pathway analysis on diatom proteomes, we here demonstrated downregulation of photosynthesis, carbon metabolism, and ribosome biogenesis in the cellular process that leads to decrease the oxidoreductase activity and affects the cell cycle of the diatom. Using PLS-DA VIP score plot analysis, we identified 15 protein biomarkers for discriminating studied samples. Of these, five proteins or gene (rbcL, PRK, atpB, DNA-binding, and signal transduction) identified as key biomarkers, induced by temperature and silicate stress in diatom metabolism. Our results show that proteomic finger-printing of *S. dohrnii* with different environmental conditions adds biological information that strengthens marine phytoplankton proteome analysis.

## Introduction

The increasing amount of anthropogenic greenhouse gas emission has resulted significant changes in the physical and chemical properties of the global ocean that have intense implications to the marine ecosystem (Pörtner et al., 2014). It is assumed that the warming ocean will enhance the nutrient stratification and modulates the ecophysiology of marine organisms (Reinfelder et al., 2000). A recent report described increasing sea surface temperature (SST), and depletion of nutrients affects the phytoplankton community resulting in 6% global biomass decrease by the end of this century (Chust et al., 2014). Further, it is reported that warming ocean will cause phytoplankton cell shifting (Lewandowska et al., 2014), cell size reduction, and grazing effects (Lindh et al., 2013; Peter and Sommer, 2013). However, the metabolic response of phytoplankton with combining environmental stress (i.e., temperature and nutrient limitation) remains unknown (Philippart et al., 2011).

The unicellular microalgae (i.e., diatoms) are the dominant phytoplankton group covering 20% of global net primary productivity and fixing 30%–50% of the inorganic carbon in the ocean; it’s equivalent to all rainforest accounts (Field et al., 1998). The macronutrients: nitrate, phosphate, and silicate are essential for the primary productivity, growth, and distribution of diatoms in the ocean (Katayama et al., 2012). Diatoms are the largest group of silicifying organisms (Otzen, 2012; Shrestha et al., 2012), and, changes in silicate concentration in the environment regulate their cell physiology and metabolism (Thangaraj et al., 2019). Earlier investigations noted during temperature fluctuation an autotroph physiological photoprotective mechanism was strongly regulated (Ruban and Johnson, 2009). As diatoms are autotroph, temperature plays a vital role in their photosynthetic mechanisms (PSII, PSI, and LHCs), and transition in temperature could alter their photosynthetic mechanisms and regulate their carbon fixation process (Dong et al., 2016).

The genus *Skeletonema* (Bacillariophyta, family: *Skeletonemaceae*) are commonly found in marine and coastal environment (Sarno et al., 2005); this genus (*Skeletonema*) has been considered a key group in diatom research due to its tropic importance to grazers, global abundance, and similar physiology of other species (Thangaraj and Sun, 2020). Among them, *Skeletonema dohrnii* is a cosmopolitan species with chain-forming cylindrical type, first identified by Sarno et al. (2005), and reported as widely distributed in temperate regions of the southern and northern hemispheres with an optimal temperature of 15–25°C (Kaeriyama et al., 2011). The occurrence and distribution of this species likely to variant as functions of environmental conditions such as temperature, nutrients, and other symbiotic organisms (Gu et al., 2012). It forms an algal blooms in Chinese coastal waters during spring season with a diameter size 3–10 microns (Gu et al., 2012) and makes a considerable impact in a coastal ecosystem.

It is predicted that temperature changes in the future will decrease diatom distribution by 10% globally and by 60% of North Atlantic and sub-Antarctic regions alone (Bopp et al., 2005). Besides, diatom provides an energy-efficient food web to support coastal fisheries (Mann, 1993). Hence, changes in diatom populations have an intense impact on the marine ecosystem and human food sources. Therefore, the continued changes in marine ecosystem by human activities could impact the future projections of mechanistic diatom response to climate change (Halpern et al., 2012).

Metabolomic profiling, transcriptomic, and proteomic approaches are powerful tools to understand the organism strategies to thrive in different environments (Darby et al., 2014; Qing et al., 2016). In the case of diatoms, their individual proteomic responses to different environments, i.e., silica limitation (Thangaraj et al., 2019) phosphorus limitation (Feng et al., 2015), nitrogen starvation (Hockin et al., 2012), iron restriction (Nunn et al., 2013), temperature stress (Dong et al., 2016), and salinity changes (Lyon et al., 2011; Kettles et al., 2014). Especially, in our recent proteomic investigation of *S. dohrnii*, between Si-deplete and replete conditions, we revealed that Si-deprivation alone led to regulate the cellular metabolism (Thangaraj et al., 2019). However, in another proteomic study on the diatom *Thalassiosira pseudonana* revealed the influence of higher temperature on the photosystem electron transport and pigment variation (Dong et al., 2016). Despite these investigations reporting significant metabolic responses to individual temperature and silicate conditions, to date, the effects of combined stressors remains unknown to predict the impact of a concomitant warming ocean and nutrient stratification.

The new method using isobaric peptide tags for relative and absolute quantification (iTRAQ) of proteins in different samples was a significant breakthrough in the qualitative and quantitative analysis of proteomics using mass spectrometry. These chemical tags attached to all peptides in a protein digest via free amines at the peptide N-terminus and on the side chain of lysine residues (Van Domselaar et al., 2005). Shotgun proteomic profiling enables us to identify proteins that are up-regulated or down-regulated under specific conditions, and this can be studied in different cell and tissue lysates. In this study, we applied mass spectrometry and iTRAQ combined proteomic profiling of the diatom *S. dohrnii* to identify and quantify the proteins to elucidate their molecular function and interactions with other proteins. Further, the multivariate statistical tool, principal component analysis (PCA), partial least squares discriminant analysis (PLS-DA) can be applied to discriminate the sample group and to identify the protein biomarkers within a given sample group.’

The primary objective of this study is to apply mass spectrometry-based proteomic profiling to identify and quantify the response of proteins to combined multiple stressors; specifically, variations in temperature and silicate, in the marine diatom *S. dohrnii.* The results are discussed in the context of diatom response mechanisms to the concomitant warming and nutrient stratification in the marine environment, and subsequent implications on the marine ecosystem, photosynthetic efficiency, and carbon fixation.

## Materials and Methods

### Chemicals and Reagents

The chemical composition of the Artificial Sea Water (ASW) is given in the supplementary section (General methods; Supplementary Table S4). Further experimental setup and bioinformatics and proteomic data analysis are shown in Supplementary Table S4 and Figure S1.

### Experimental Design: Algal Culture and Multiple Climate-Related Variables

A diatom *S. dohrnii* used in this study was isolated from the Yellow Sea coastal waters and cultured in the f/2 medium (Guillard and Ryther, 1962) in the laboratory by applying 12 h:12 h light:dark cycle using cool white fluorescent light, with a light irradiance of 170 μmol photons m^2^ s^–1^. To ensure axenic condition, 0.05 mg ml^–1^ of the antibiotic gentamicin, 0.8 mg ml^–1^ of streptomycin, and 1.6 mg ml^–1^ of penicillin was used according to published protocols (Kobayashi et al., 2003; Bahulikar and Kroth, 2008). We applied a semi-continuous culturing approach to study the impact of temperature (T) and silicate (Si) conditions on the proteome using iTRAQ based proteomic analysis.

The experiment, *S. dohrnii* was maintained in two different temperature conditions [low temperature (15°C) and high temperature (25°C)] in the Aquil, synthetic ocean water/Artificial Sea Water (SOW/ASW), medium with different concentrations of silicate [low silicate (0.2 ml/L) and high silicate (2ml/L)]. In order to prevent silicate contamination from the bottles, and therefore extra silicate utilization of *S. dohrnii* during the experiment, cells were grown in Nalgene, Reusable Baffled Erlenmeyer Culture flasks made of polycarbonate. The four different sample conditions (with replication) were as follows: HTHS (T: 25°C and Si: 2 ml/L), HTLS (25°C and 0.2 ml/L), LTHS (15°C and 2 ml/L), and LTLS (15°C and 0.2 ml/L). Cultures were maintained for up to five generations to ensure cell acclimation. Cultures were harvested 21 days after the start of the experiment for quantitative proteomic analysis. At each generation, less than 15% of cells were obtained during mid-exponential stage (day 4) and transferred to inoculate fresh 1 L cultures.

The reproducibility of each condition was tested using three independent triplicate experiments. Cell growth and density were analyzed using a Qiujing hemocytometer and inverted microscope AE 2000 (Motic Group Co., Ltd., China). The cell density was calculated as follows: CD = (*N*/80) Å∼400 Å∼104, where CD is the cell density, *N* is the cell abundance counted in 80 grids on the slide. After five generations, the acclimated cells were harvested by centrifugation at 4000 Å∼10 min at 4°C for proteomics analysis.

### Pigment Analysis

For pigment analysis, 100 ml per each sample were collected and filtered using GF/F filters, and the membrane was flash-frozen with liquid nitrogen and stored at −80°C until further analysis. Pigments were extracted using 3 ml methanol, then ultrasound was applied in an ice bath for 30 s, and kept at −20°C for 1 h. Initially, the extracted samples were filtered through a 0.22 μM membrane filter to remove the detritus and mixed with 28 mmol/L of tetrabutylammonium acetate (TBAA). Subsequent grinding, centrifugation, and filtration steps were performed following Zapata et al. (2000). All the procedures were done under subdued light.

Pigment quantification was performed using HPLC (Agilent 1260 Series Infinity, United States) following Zapata et al. (2000). The HPLC system was equipped with an Eclipse XDB C8 column (150 mm × 4.6 mm, 3.5 μm particle size), an Agilent diode array detector with wavelength range 350–750 nm (absorbance at 440 nm) and the ChemStation software (Agilent Tech). Mobile phase A comprised 28 mM TBAA (pH = 6.5): methyl alcohol = 3/7 (v/v) and reagent B: 100% methyl alcohol. Solvents were mixed using linear gradients along the following time program: (0 min: 80%A, 20%B), (32 min: 25%A, 75%B), (48 min: 5% A, 95%B), (56 min: 80%A, 20%B), (60 min: 80%A, 20%B). The flow rate was set 1.0 ml/min, and the temperature of the column oven was at 45°C.

In the present study, all the pigments were identified and quantified using the pigment standard, which was purchased from DHI Water & Environment, Hørsholm, Denmark. The following pigments were detected and quantified: Chlorophyll *c*3 (Chl *c*3), Chlorophyll *c*2 (Chl *c*2), Peridinin (Perid), Fucoxanthin (Fuco), Diadinoxanthin (Diadino), Diatoxanthin (Diato), Chlorophyll *a* (Chl *a*), and β-carotene (β-car). Generated peaks were integrated by using Agilent software, but all peak integrations were checked manually and corrected when necessary and quantified, using the standard external method. Diatom pigment were identified using the comparison of chromatographic and recorded spectral data with standard pigments. For all the physiological parameters, multiple analysis of variance (MANOVA) was performed using the R basic package to determine any significant changes among four different samples (HTHS, HTLS, LTHS, and LTLS).

### Protein Preparation and Digestion

One liter of culture from each sample was collected through a 2 μM filter and subsequently suspended in 10 ml medium using 15 ml centrifuge tubes for protein preparation (Du et al., 2014). The resulting cell pellets were then suspended in a lysis buffer (8 M urea, 40 mM Tris–HCl or TEAB with 1 mM PMSF, 2 mM EDTA, and 10 mM DTT, pH 8.5). The mixture of samples was placed into a tissue lyser for 2 min at 50 Hz to achieve cell lysis, and then centrifuged at 25,000 × *g* for 20 min at 4°C. The supernatant was then transferred into a new tube; samples were reduced with 10 mM dithiothreitol (DTT) at 56°C for 1 h and alkylated by 55 mM iodoacetamide (IAM) in the dark at room temperature for 45 min to block the cysteine residues of the proteins. Following centrifugation (25,000 × *g*, for 20 min at 4°C), the supernatant containing proteins was quantified by the Bradford assay method (Kruger, 2009). The protein solution (100 μg) with 8 M urea was diluted four times with 100 mM TEAB and then incubated for at least 1 h at −20°C, followed by centrifugation of the precipitate. The pellet washed with 90% ice-cold ethanol, and the supernatant was removed, and the pellet resuspend in a buffer (8 M urea, 2 M thiourea, 2% SDS, and 40 mM Tris). Trypsin Gold (Promega, Madison, WI, United States) was used for the protein digestion with a ratio of: trypsin = 40:1 at 37°C overnight. After trypsin digestion, peptides were desalted with a Strata X C18 column (Phenomenex) and vacuum-dried according to the manufacturer’s protocol for 8-plex iTRAQ (Applied Biosystems, Foster City, CA, United States).

### Analytical Procedure and Peptide Labeling

Eight samples consisting of two biological replicates for four-time points were labeled with different iTRAQ tags. Briefly, peptides were labeled with iTRAQ reagents 113 and 114 for HTHS samples; 115 and 116 for HTLS samples; 117 and 118 for LTHS samples; and 119 and 121 for LTLS samples. The labeled peptide blends were pooled and dried through vacuum centrifugation and fractionated. All solvents used for high-performance liquid chromatography (HPLC) was HPLC grade (Sigma-Aldrich), and the H_2_O was Millipore Milli-Q PF filtered. The peptides, were separated on a Shimadzu LC-20AB HPLC Pump system coupled with a high pH reverse phase column (Gemini C_18_ 5 μM, 4.6 mm × 250 mm), The peptides were reconstituted to HPLC separation with following mobile phase (A) 5% ACN, (B) 95% H_2_O (adjusted pH to 9.8 with 2 ml of NH_3_), sample input and acquisition; 2 ml/min flow rate and 1 ml/min injection volume. Crude peptide compound elution was monitored by measuring UV absorbance at 214 nm, and the 40 fractions were collected every 1 min. All the eluted peptides were combined as 20 fractions and vacuum-dried for further process. Furthermore, each fraction was resuspended in buffer A (2% ACN and 0.1% Formic Acid in H_2_O) and then centrifuged at 20,000 × *g* for 10 min and independently subjected to HPLC separation (LC-20AD nano-HPLC instrument, Shimadzu, Kyoto, Japan) using C_18_ column (inner diameter 75 μm). Sample input and acquisition; 300 nl/min flow rate and 1 μl injection volume for 8 min, the 35 min gradient was run at 300 nl/min starting from 8% to 35% of buffer B (2% H_2_O and 0.1% FA in ACN), followed by a 5 min linear gradient to 80% solution B, maintenance at 80% solution B for 4 min, and return to 5% in 0.1 min and equilibrated for 10 min.

### LC-MS/MS Proteomic Analysis

Liquid Chromatography and Mass Spectrometry (LC-MS) analysis of diatom peptide were performed on LC-20AD (Shimadzu, Kyoto, Japan) using C_18_ column (size 75 μm). The LC-MS data were acquired in positive ion mode of data independent acquisition (DIA) within a selected mass range of 350–1500 *m/z*. Based on the intensity in MS1 survey, as many as 30 production scans were collected if beyond a threshold of 120 counts per second (counts/s) and with charge-state 2+ to 5+ dynamic exclusion was set for 1/2 of peak width (12 s). For MS data acquisition, the collision energy was adjusted to all precursor ions for collision-induced dissociation and the Q2 transmission window for 100 Da was 100%.

### Bioinformatics and Proteomic Data Analysis

All the mass spectral data were processed using the ProteoWizard software-msConvert with default parameters for generating peak list. The data alignment was performed with Analyst QS 2.0 software (Applied Biosystems/MDS SCIEX). Further, protein identification and quantification were achieved using Mascot 2.3.02 (Matrix Science, London, United Kingdom) (Charbonneau et al., 2007). The greatest extents of the iTRAQ reporter ions mimic the relative abundance of the proteins in the samples. TripleTOF 5600 mass spectrometer with high mass accuracy resolution (less than 2 ppm) was used in this study for peptide identification. Other identification parameters used included: fragment mass tolerance: ±0.1 Da; mass values: monoisotopic; variable modifications: Gln->pyro-Glu (N-term Q), oxidation (M), iTRAQ8plex (Y); peptide mass tolerance: 0.05 Da; max missed cleavages: 1; fixed modifications: carbamidomethyl (C), iTRAQ8plex (N-term), iTRAQ8plex (K); other parameters: default. For iTRAQ quantification, the peptide for quantification was automatically selected by the algorithm to calculate the reporter peak area (using default parameters in the Mascot Software package). The acquiring data was auto bias-corrected to get rid of any differences imparted due to the unequal mixing during combining differently labeled samples. Proteins with the 1.2-fold change between each different sample and a *p*-value of statistical evaluation less than 0.05 were determined as differentially expressed proteins (DEPs). All proteins were identified by MS/MS ion search using Mascot version 2.3.02, mass tolerance 0.05 Da. The students’ *t*-test was performed using the mascot 2.3.02 software. Briefly, a protein ratio is reported in the boldface if it is significantly different from unity. The comparison test is:

$$\left|X-\mu \right|\leq t^{*}\,\frac{S}{\sqrt{N}}$$

If this dissimilarity is real, then there is no important difference at the stated sureness level. Further, *N* is the number of peptide ratios, *S* is the standard deviation, and *x* the mean of the peptide ratios, both numbers calculated in log space. The metabolic pathway analysis of the identified proteins was conducted according to the KEGG Pathway Database (Kanehisa and Goto, 2000; Kanehisa et al., 2016). The Gene Ontology (GO) and Cluster of Orthologous Groups of proteins (COG) analyses (http://www.geneontology.org) were performed according to the method reported in the early literature (Unwin, 2010). The enhancement of differentially regulated proteins in GO terms was carried out using the following formula:

$$P=1-\sum_{i=0}^{m-1}\frac{\left(\begin{array}{c} M\\ i \end{array}\right)\,\left(\begin{array}{c} N-M\\ n-i \end{array}\right)}{\left(\begin{array}{c} N\\ n \end{array}\right)}$$

where *N* is the number of all proteins with GO annotation information, *n* is the number of the differentially regulated proteins with GO annotation information, *M* is the number of proteins with a given GO term annotation, *m* is the number of the differentially regulated proteins with a given GO term annotation. The GO terms with a *p*-value of less than 0.05 were considered as enriched GO terms by the stress-responsive proteins which may be involved in temperature and silicate induces stress. The multivariate analysis model PCA and PLS-DA analysis was performed with R package (version 1.78).

## Results

### Physiological Changes

**FIGURE 1:**
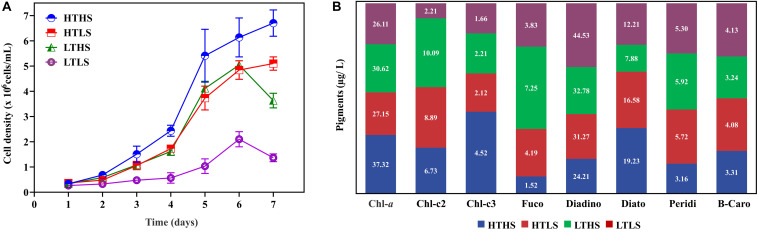
**(A)** Cell density in four different conditions. HTHS, high silicate high temperature; HTLS, high silicate low temperature; LTHS. low-temperature high silicate; LTLS, low-temperature low silicate. The error bars represent the standard errors from triplicate measurements and **(B)**, the observed pigment composition in *Skeletonema dohrnii* under different stress conditions.

Cells were cultured in four different environmental conditions as described in the Materials and Methods. The results of cell density in each condition are shown in Figure 1A. Different cell responses to each environment were evident from day 2. Notably, during the exponential phase (day 5) the cell density in HTHS increased drastically, whereas it decreased in HTLS and LTHS. Both silicate stressed HTLS and temperature stressed LTHS treatments showing a similar physiological regulation (Figure 1A). The cell density of LTLS in the exponential phase (1.03 × 10^6^) was three times lower than the HTHS sample (4.47 × 10^6^), and two times lower than HTLS (3.2 × 10^6^). Further, once cells reach the peak density (day 6), cells in the HTHS and HTLS treatments-maintained growth rates whereas, in the LTHS and LTLS conditions, growth declining rapidly.

### Quantification of Pigments

The pigment analysis has been carried out using HPLC to understand the impact of abiotic stress on the pigment composition of the *S. dohrnii* (Figure 1B). Overall, eight pigments were identified from each sample including chlorophyll-a (Chl-a), fucoxanthin, diadinoxanthin, and β-carotene. Among all the pigments in HTHS, Chl-a was the highest (37.32 μg/L) followed by the diadinoxanthin (24.21 μg/L). In the remaining samples, the diadinoxanthin was the highest followed by Chl-a, whereas, the other accessory pigments such as peridinin, fucoxanthin, and β-carotene were lower in the composition.

### Protein Identification

Using i-TRAQ-labeled LC-MS/MS analysis from *S. dohrnii*, a total of 3,70,713 spectra were identified from all samples; a total of 3359 peptides and 1803 proteins were identified with 1% FDR. Several studies reported similar results using the iTRAQ method on *T. pseudonana* 1831 (Du et al., 2014), and 1850 (Nunn et al., 2013) from silicate and iron-deplete and replete conditions respectively. However, this result varies with other proteomic studies on diatoms between higher and lower light 4183 proteins (Dong et al., 2016) multiple nutrient stress (Si, N, P) 3798 (Chen et al., 2018) Fe deprivation, 1204 (Cohen et al., 2018) nitrogen depletion, 1043 (Longworth et al., 2016), phosphorus stress 1264 (Dyhrman et al., 2012), and phosphate limitation, 1151 (Lin et al., 2017) and in our previous study *S. dohrnii* 1768 proteins (Thangaraj et al., 2019), respectively. The differential protein expression identified in this study between groups showed that the repeatability between the replicates was acceptable with 1.2-fold change, mean CV of 0.16, and statistically significant in between the group comparisons (*p* < *0.05*). The distribution of protein mass, peptide length distribution, unique peptide number distribution, spectral number, and coverage distribution is shown in Supplementary Table S4 and Figure S2.

### Quantification of Identified Proteins

**FIGURE 2:**
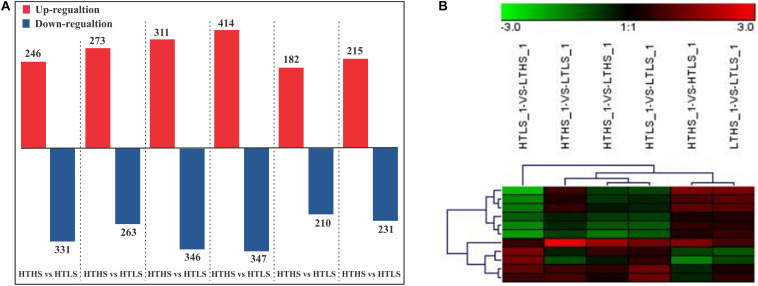
**(A)** Bar plot of differentially expressed protein in each pairwise comparison and **(B)** the differently expressed proteins (down- and up-regulation). *X*-axis, names of the comparison groups; *Y*-axis, the number of the differentially expressed protein. Red column, up-regulation protein; Green column, down-regulation protein.

In our study, a total of 1803 proteins were identified (See Supplementary Table S1) in response to changes in temperature and silicate concentrations in diatom *S. dohrnii.* Further, among the overall quantified proteins, 536 differently expressed proteins were identified between a higher temperature (HT) and lower temperature (LT), of which 263 were downregulated (Supplementary Table S2) and 273 up-regulated (Supplementary Table S3). Interestingly, 577 differentially expressed proteins were distinguished between higher silicate (HS) and lower silicate (LS), of which diatom 331 proteins down-regulated (Supplementary Table S2) and 246 proteins up-regulated (Supplementary Table S3). The comparison of differently expressed proteins between each sample groups is given in Figure 2A. The differently expressed proteins also have been identified using the volcano plot analysis method (Supplementary Table S4 and Figure S3). In this study, the maximum number of down-regulated proteins was observed in group HTHS versus HTLS and up-regulated proteins in group HTHS versus LTHS. The differentially changed proteins identified in our experiments were grouped into different sample groups based on cluster analysis using Euclidean distance method and hierarchical algorithm (Figure 2B and Supplementary Table S4 and Figure S4). To understand how unique or similar of proteins identified from diatom *S. dohrnii*, those were compared with other diatoms (See Supplementary Table S4A). Among all the maximum number proteins (1030) and (627) in *S. dohrnii* was similar with *T. pseudonana*, *T. oceanica* respectively. Additionally, only fewer proteins (12) and (5) of *S. dohrnii* was similar with *S. costatum* and *S. marinoi* despite the same genus.

### Functional Annotation of Temperature and Silicate Responsive Proteins

Gene Ontology (GO) was analyzed to identify significant biological changes of the differently abundant protein of diatom *S. dohrnii* to changes of temperature (15 and 25°C), and silicate (0.2 and 2 ml) in the cultures. A total of 43 functional groups were identified (Supplementary Table S4 and Figure S5), of which molecular function accounted for 11 GO terms, cellular component accounted for 13 GO terms, and biological process accounted for 19 GO terms. The four-primary molecular function of the GO terms was a catalytic activity, binding, structural molecular activity, and transporter activity. In cellular component GO annotation, cell, cell part, organelle, macromolecular complex, and membrane part was the top cellular components GO categories. In the biological process GO annotation, more than 85% of the proteins were annotated with the metabolic process, i.e., cellular process, single-organism process, and response to a stimulus. Further, the Gene Ontology enrichment analysis indicated that many processes were associated with responses to changes of temperature and silicates in diatoms, including photosynthesis (*p* < 0.01), carbon metabolism and carbon fixation in photosynthetic organisms (*p* < 0.02), glycolysis or gluconeogenesis (*p* < 0.02), oxidative phosphorylation (*p* < 0.04), biosynthesis of amino acids (*p* < 0.01), and biosynthesis of secondary metabolites (*p* < 0.01).

### COG Annotation for All Identified Proteins

**FIGURE 3:**
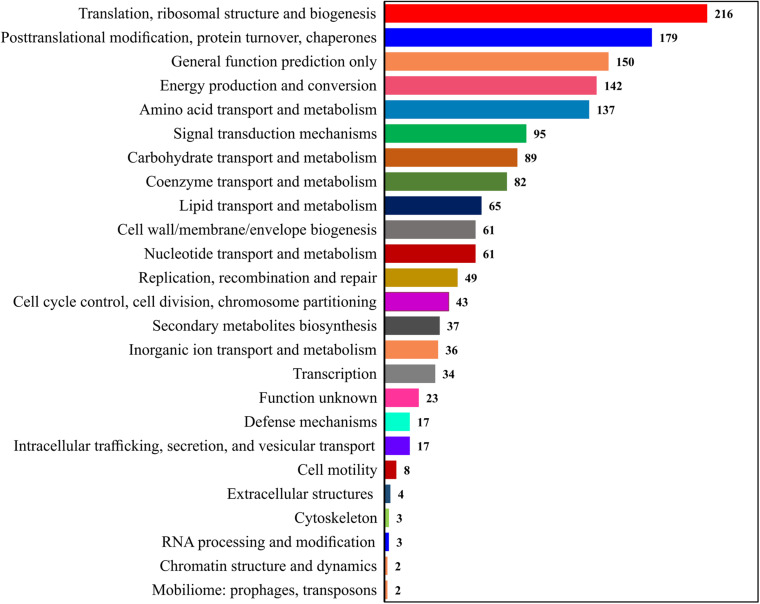
Representation of the protein sets from complete genomes in the COGs.

The Clusters of Orthologous Groups of proteins (COGs) annotation was applied to classify proteins from the sequenced genomes using orthologs concept (Figure 3). The COGs were classified into 25 functional categories according to Riley (1993), showing that the maximum of 216 proteins involved in translation and ribosomal process, followed by 179 proteins in posttranslational modification, 142 proteins in energy production and conversion, 137 proteins in amino acid transport, 95 proteins in signal mechanisms, 89 proteins in carbohydrate transport, and 82 proteins in coenzyme transport and metabolisms.

### Multivariate Statistical Analysis

**FIGURE 4:**
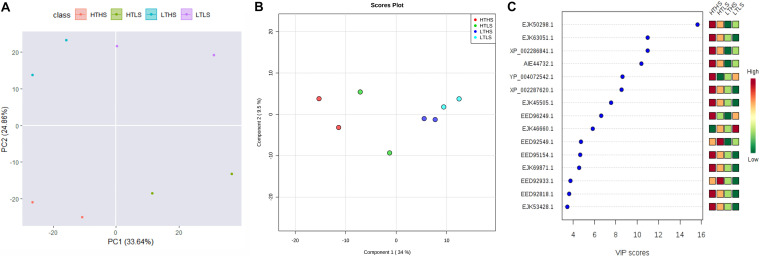
The multivariate statistical analysis score plots on *S. dohrnii* proteins quantified across all eight samples. **(A)** Scatter plot of the first two principal component analysis (PCA) of diatom *S. dohrnii* iTRAQ data. **(B)** PLS-DA score plot analysis obtained from diatom *S. dohrnii.* **(C)** Variable Important in Projection (VIP Score plot) the estimation of important variables in the projection used in PLS-DA model.

An unsupervised Principal Component Analysis (PCA) was applied to discriminate the samples of different environmental conditions using the proteins ratio and mass spectral variables of each culture. In PCA analysis, the samples were clearly discriminated four different cultures or environmental conditions based on the up- or down-regulation of proteins (Figure 4A). The supervised Partial Least Square Analysis model (PLS-DA) was also applied to emphasize the variation in dataset and used to predict Variable Importance Projection (VIP) score to identify the discriminating the samples based on the environmental conditions. The PLS-DA analysis showed similar discrimination among samples HTHS, HTLS, LTHS, and LTLS (Figure 4B). Even with the two biological replicates (*n* = 2) per group that is employed in our study, the VIP score of the top 15 proteins is higher than 3 (Figure 4C). The first 15 proteins (protein biomarkers) that explain the differences between the four samples groups (HTHS, HTLS, LTHS, LTLS) are given in Table 1.

**TABLE 1:** The protein chemo markers responsible for the discrimination of four sample groups.

**Gene name**	**Protein ID**	**Name**	**GO function**	**Mass**	**Protein coverage**
THAOC_30748	EJK50298.1	ERCC4 domain-containing protein	DNA binding/nuclease activity	73,282.17	0.018
THAOC_16311	EJK63051.1	hypothetical protein	Protein phosphorylation	71,182.03	0.083
THAPSDRAFT_31562	XP_002286841.1	Predicted protein	Plasma membrane	9040.44	0.163
THAPSDRAFT_233	XP_002287620.1	Predicted protein	—	29,947.35	0.088
rbcL	AIE44732.1	Ribulose-1,5-bisphosphate carboxylase (chloroplast)	Reductive pentose-phosphate cycle	24,004.25	0.363
THAOC_35879	EJK45505.1	Protein YIF1B	Endoplasmic reticulum to Golgi vesicle-mediated transport	37,548.29	0.038
atpB	YP_004072542.1	ATP synthase CF1 subunit beta (chloroplast)	ATP synthesis coupled proton transport	51,155.44	0.319
THAOC_08831	EJK69871.1	PRK domain-containing protein	Carbohydrate metabolic process	20,838.29	0.267
THAPSDRAFT_2247	EED95154.1	Predicted protein	—	14,816.54	0.108
THAPSDRAFT_31510	EED96249.1	Fe-S_biosyn domain-containing protein	Iron–sulfur cluster assembly/protein maturation	18,689.72	0.07
PRK1	EED92818.1	Phosphoribulokinase (PRK)	Phosphorylation	42,515.47	0.072
THAOC_34663	EJK46660.1	PAS domain-containing protein	Signal transduction/protein kinase	24,629.05	0.057
THAPS_10777	XP_002295511.1	Hypothetical protein	Integral component of membrane	43,249.56	0.029
THAOC_27143	EJK53428.1	Hypothetical protein	—	33,381.81	0.026
ANS1	EED92933.1	Asparagine synthase	Asparagine biosynthetic process	66,174.51	0.073

*All proteins were identified by MS/MS ion search using Mascot version 2.3.02, mass tolerance 0.05 Da.*

### Protein Involvement in Metabolic Pathways

To further investigate the biological function of differentially expressed proteins from diatom *S. dohrnii*, these DEPs were annotated to the KEGG pathways. In these six groups, the maximum 127–241 proteins involved metabolic pathways was the most significant term among biological processes, following that biosynthesis of amino acids (31–73 proteins), carbon metabolism (41–68 proteins), glycolysis or gluconeogenesis (13–27 proteins), and photosynthesis (9–25 proteins) were the most enriched KEGG pathways (Supplementary Tables S4B–G).

**FIGURE 5:**
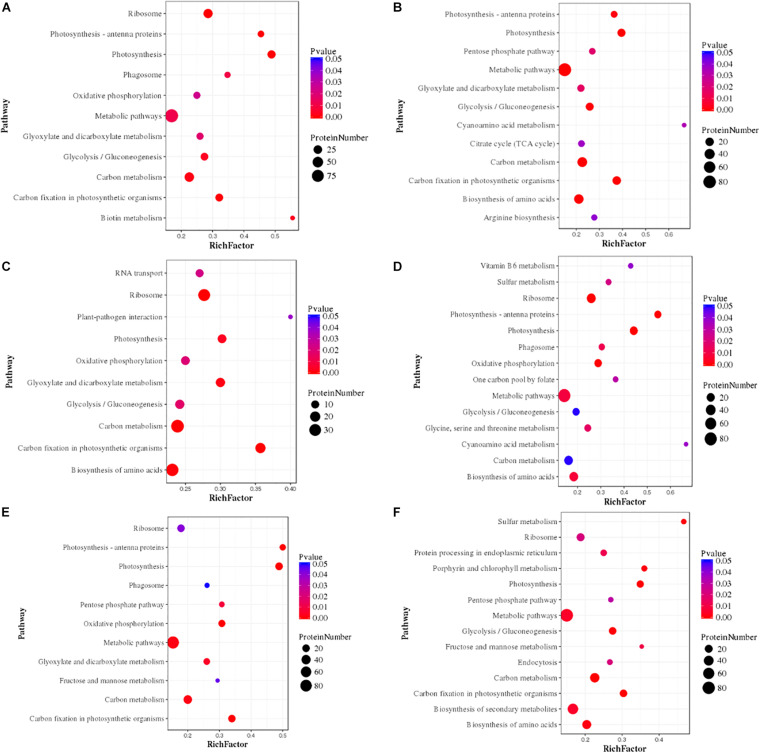
Pathway enrichment analysis of differentially expressed proteins in each pairwise. In the figure greater rich factor means greater intensiveness and *p*-value ranges from 0 to 1, and less *p*-value means greater intensiveness. **(A)** (HTHS-HTLS), **(B)** (HTHS-LTHS), **(C)** (HTHS-LTLS), **(D)** (HTLS-LTHS), **(E)** (HTLS-LTLS), **(F)** (LTHS-LTLS).

We proposed the top most influential or significant pathways (*p* < 0.05) of temperature and silicate response to *S. dohrnii* using pathway enrichment analysis of each condition (Figure 5). The results indicated that in HTHS versus HTLS conditions the differentially expressed proteins were predominately enriched in ribosome metabolism, 68 proteins, (*p* < 0.0000053), followed by 68 proteins in carbon metabolism (*p* < 0.03), and 24 proteins in photosynthesis metabolism (*p* < 0.05). In HTHS versus LTHS, 180 proteins were enriched in 90 pathways in the KEGG database, represents, 41 DEPs in ribosome pathways (*p* < 0.001), 20 DEPs in photosynthesis metabolism (*p* < 0.001), and 22 DEPs in carbon fixation metabolism (*p* < 0.04). In group HTHS versus LTLS, 204 DEPs were enriched in 96 metabolic pathways in the KEGG database. Further, pathway enrichment analysis shows, with 32 proteins alteration in carbon fixation metabolism, (*p* < 0.001), 68 proteins in carbon utilization metabolism (*p* < 0.03), and 64 proteins in ribosome pathway (*p* < 0.0001), were the significant metabolism response to this condition. Similarly, the comparison between HTLS versus LTHS sample, shows, 61 DEPs in ribosome pathways, (*p* < 0.006) followed by 19 DEPs in pigment metabolism (*p* < 0.00002), and 73 proteins amino acid biosynthesis were the most influenced biological pathways in *S. dohrnii*. In group HTLS versus LTLS, 127 DEPs were enriched in 93 pathways in the KEGG database. Among all, carbon metabolism was the most represented pathway (*p* < 0.06), followed by thiamine metabolism (*p* < 0.01), and vitamin B6 metabolism (*p* < 0.04). Whereas, DEPs involved in the amino acid biosynthesis (*p* < 0.001), and carbon metabolism (*p* < 0.06) were the most altered pathways of *S. dohrnii* in LTHS versus LTLS group.

## Discussion

In this study, we demonstrated the application iTRAQ, bioinformatics analysis (GO, COG, VIP score plot), and prediction of protein-enriched pathway (KEGG pathway) in quantitative proteome profiling of the ecologically important marine diatom *S. dohrnii.* The results of this study demonstrated that phenotypic plasticity of diatom proteome could be widely regulated of tolerance limits in response to global warming. The changes of proteome profile and associated cellular functions in different environmental conditions of the diatom *S. dohrnii* in response to climate change and impacts on marine ecosystem are discussed here.

### Physiological Changes

Elevated temperature under conditions of nutrient availability led to higher intrinsic growth rates, promoting enhancement in energetic, metabolic, and gene expression that induces smooth cell cycle and cell division in diatoms. In addition to warming, to cope with nutrient limitation diatoms could modify several metabolic pathways. For instance, diatoms responded to phosphate limitation expressed modification in the glycolysis pathway and induce a higher level of lipids and primary triacylglycerols (Longworth et al., 2016). During the phosphate limitation diatom replaces phospholipids with non-phosphorous membrane lipids to reduce the demand of phosphate (Van Mooy et al., 2009) and cope up with nutrient limitation. *S. dohrnii* may have a similar strategy in HTLS condition to cope with the warming ocean with under silicate limitation.

It was proposed that acetyl-CoA metabolism in diatom played a key role when diatom grew in lower temperature. A supply of acetyl-CoA to fatty acid and Branched-Chain Amino Acid (BCAA) metabolism will reduce the cell division rates (Cai et al., 2011; Pietrocola et al., 2015; Lin et al., 2017) while excessive cytosolic acetyl-CoA resulting in the over acetylation of cytosolic proteins and consequent inhibition of enzymatic activity (Weinert et al., 2014). Therefore, it is proposed that invested pathways in fatty acid and BCAA play a key during the LTHS adaptation. Moreover, in diatoms cell division and cell cycle consist of light-dependent and light-independent segments (Ashworth et al., 2013) which regulates diatom cell cycle G1 and G2/M phases (Huysman et al., 2014). However, in some instances cell division determined by light phasing in diatom can be regulated and by nutrient addition, suggesting nutrient control of the cell cycle is increased during the temperature variation (Olson and Chisholm, 1983). Besides, diatom treated lower temperature, and lower silicate expressed lower protein ratio, which could decrease membrane fluidity and can affect the compensatory investment of photosynthesis proteins and biosynthesis of fatty acids. This eventually induces cell cycle arrest at the G1 phase, which has been linked with silica requirement for DNA replication (Huysman et al., 2010) could be the reason for lower cell growth and replication in LTLS condition.

### Mechanisms Underlying Tolerance to Temperature and Silicate – Proteomics Perspective

Marine organisms responses to various environmental stresses, i.e., warming, ocean acidification, low salinity, lower silicate, doxycycline at proteomics level have been studied and reviewed (Allen et al., 2008; Clement et al., 2017; Jian et al., 2017; Tomanek, 2011). The iTRAQ based proteomic profiling revealed a systematic temperature tolerance mechanism of marine diatom. This study reveled the down-regulation of various proteins involved in the metabolic process in response to the changes in temperature and silicate level. Similarly, molecular catalytic activity process also appears to be speeded up in response to a change of temperature and silicate. The rearrangement of cellular process in energy production and conversion, biogenesis, and amino acid transport and metabolism were known to be the vital physiological adjustments in marine organisms undergo due to the effect of climate change and ocean acidification (Dupont et al., 2012; Stumpp et al., 2012; Jian et al., 2017). The effect noticed in these metabolic process pathways appears to be necessary for marine diatom because it could allow them to allocate more bioenergy for stress acclimation during higher temperature with less silicate for cell wall formation and cellular process.

Similarly, differential expression of 293 proteins involved in diatom *S. dohrnii* tissue metabolism, cell division and cycle control (43 proteins), translation and ribosomal structure (216 proteins), transcription (34 proteins) signal transduction mechanisms (95 proteins), appears to have significant role during the changes of temperature and silicate concentration at cellular level. Marine organisms including larvae and adults, tend to elevate lipid and carbohydrate metabolism to meet the higher bioenergetics demands of temperature stress, but with a physiological cost, reduced the expression of proteins involved in growth and repair (three proteins) and immune system process (eight proteins). These results indicate the presence of systematic temperature tolerance mechanisms in diatom *S. dohrnii.* The response of marine diatom *S. dohrnii* to changes of temperature and silicate at proteomics level in our study is consistent with earlier investigations (Jian et al., 2017; Thangaraj et al., 2019). The functional role of some of these differentially expressed proteins and associated pathways are discussed here.

### Down-Regulation of Photosynthesis

**FIGURE 6:**
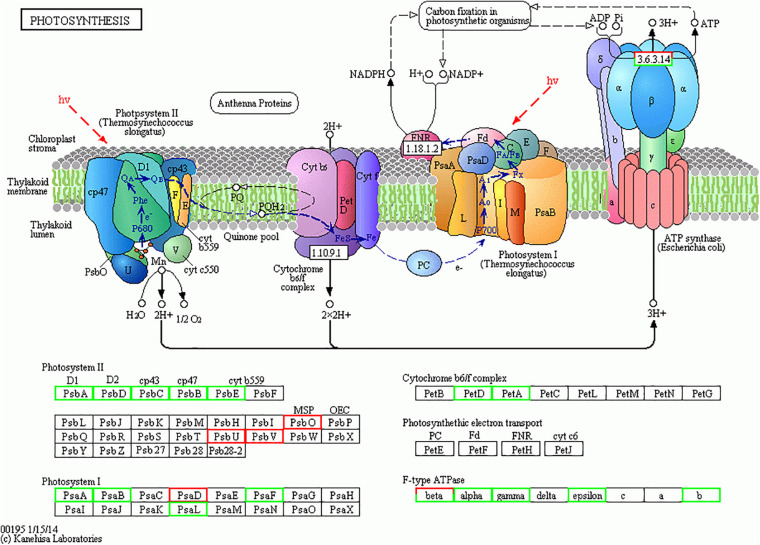
The photosynthesis pathway map for *Skeletonema dohrnii* during the changes of temperature and silicate (HTHS vs. HTLS). The down-regulated proteins were shown in light green color boxes and up-regulated proteins are given in red color. The comprehensive information of all the regulated proteins associated with photosynthesis metabolism are given in Supplementary Tables S2, S3. This image is obtained from KEGG website (http://www.kegg.jp/kegg/kegg1.html) with copy right permission.

Earlier investigation noted depletion of nitrogen on *T. pseudonana* (Jian et al., 2017) and low iron supply to *Phaeodactylum tricornutum* (Allen et al., 2008) causing significant changes in the photosynthesis process. Similarly, in our study, the higher temperature and silicate limitation lead to down-regulation of photosynthesis in diatom *S. dohrnii* (Figure 6). Based on the KEGG pathway analysis 20 photosynthesis-related proteins associated with changes of temperature and silicate (HTHS vs. HTLS), from which 16 proteins were downregulated including photosystem II protein (PsbA, PsbD, PsbC, PsbB, PsbE, PsbH), photosystem I proteins (PsaA, PsaB, PsaF, and PsaL) cytochrome (PetA, PetC, PetD), and including proton-transporting ATP synthase activity and regulate rotational mechanism proteins (F-type ATPase α, γ, and β). Four proteins were upregulated (red color) photosystem I and II proteins (PsbO, PsbV, PsaD) and F-type ATPase (β). PsaB and PsaC were two photosystem II protein binds chlorophyll and supports catalyze the primary light-induced photochemical processes of PSII (Zhang et al., 2015). PsbA, PsbB, and PsbC are three subunits assembled in photosystem II with PsbA acting as the reaction center protein, and PsbB and PsbC responsible for light-harvesting (Barber, 2002). The upregulated protein PsbO responsible for the oxidation–reduction process and PsaD protein involves in ferredoxin-binding and form complexes with ferredoxin and ferredoxin-oxidoreductase in photosystem I (PSI) reaction center of chromoplast cells. The higher abundance of downregulated proteins indicated decreased photosynthetic carbon fixation and energy production, which may contribute to reducing growth rate similar results was observed on macroalgae (Fan et al., 2018). In our experimental analysis (HTLS vs. LTHS), a total of 14 proteins were upregulated such as photosystem proteins I and II (PsbA, PsbB, PsbF, PsbV, PsaJ) and F-type ATPase (i.e., α, β, γ, and δ). It binds chlorophyll and helps catalyze the primary light-induced photochemical processes of PSII. In diatoms, protein PsbF is stimulating electron transfer, heme-binding or iron-binding process. Four proteins were downregulated (HTLS vs. LTHS), like PsbU, PetA, PetF, PetH, and α. In diatom *S. dohrnii* protein PetH involved in molecular function, particularly oxidoreductase activity. In a low-temperature experiment (LTHS vs. LTLS), KEGG analysis reveals that a total of 16 downregulated proteins were involved in the photosynthetic pathway and one upregulated protein PsbO serve as oxygen-evolving enhancer protein and regulate the oxidation–reduction process. Results indicated that both photosynthesis and other metabolic pathways were significantly associated with proteomic alteration of higher temperature and deprivation of silicate. Our findings revealed that different molecular metabolism of diatom *S. dohrnii* could be temperature and silicate dependent, specifically during with higher temperature and silicate can induce expression of upregulated proteins like photosystem I and II and could increase the growth of diatoms through photosynthesis and metabolic pathways. By contrast in high temperature and low silicate level, the photosynthetic proteins are down-regulated and reducing the planktonic metabolism, photosynthesis process, and ATP formation in diatoms, resulting the subsequent impact of zooplankton biomass and fishery production. In associated with photosynthesis metabolic regulation, the proteins regulate the light-harvesting process (pigment metabolism) are given in Supplementary Table S4H. In *S. dohrnii*, the photosynthetic pigments fucoxanthin is bound along with chlorophyll, it encompasses the light-harvesting from solar energy and its involvement in the early stages of the photosynthesis process (Thangaraj et al., 2020). In this study, fucoxanthin and chlorophyll relevant proteins were mostly downregulated with higher temperature and lower silicate concentration (HTHS vs. HTLS) and upregulated in lower temperature with higher silicate concentration (HTLS vs. LTHS), the above results suggest that the lower silicate concentration reduced the photosynthetic efficiency compared to another environmental parameter like temperature.

### Photosynthesis and Carbon-Fixation-Related Genes Decreased

**FIGURE 7:**
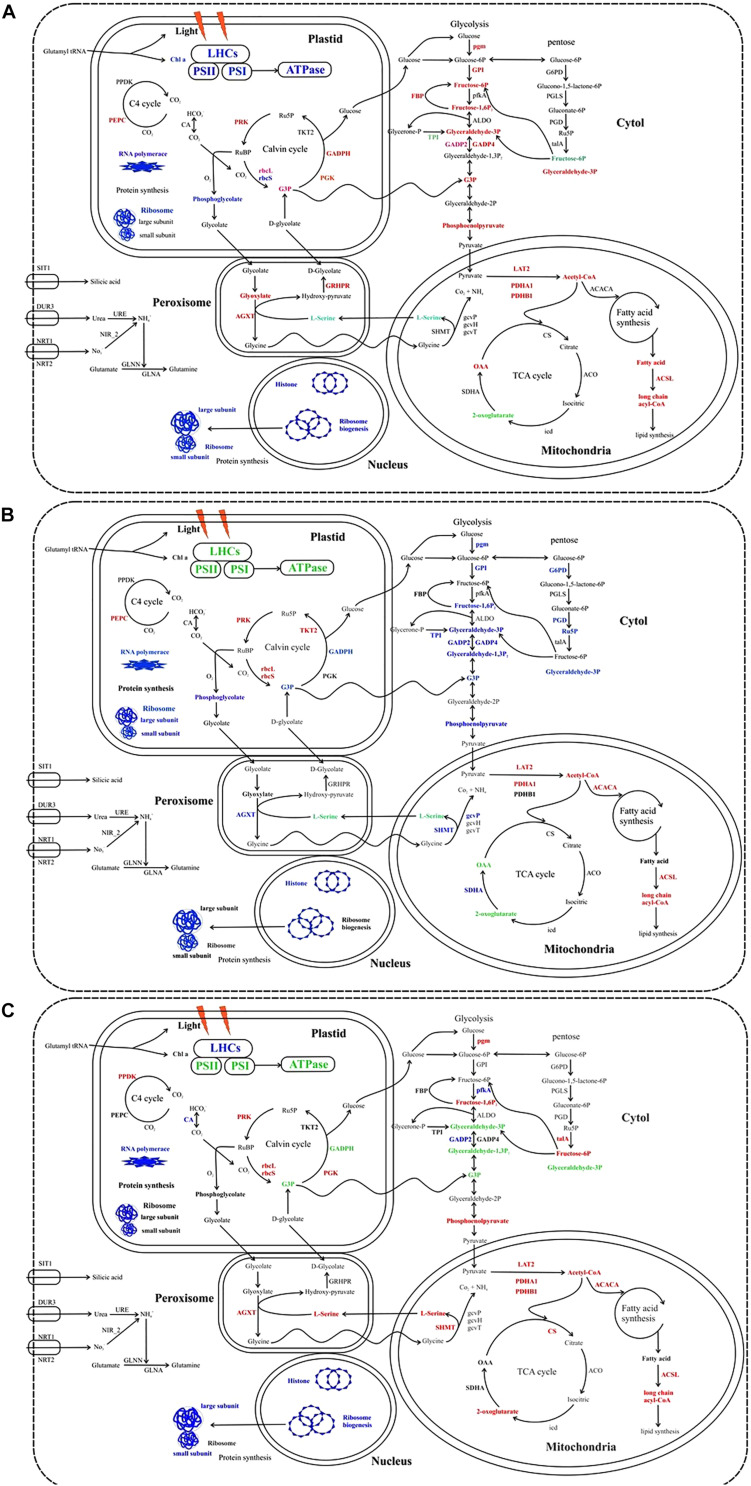
Cellular metabolic pathways and processes influenced by the different stress condition in *S. dohrnii.* **(A)** HTHS versus HTLS; **(B)** HTHS versus LTHS; **(C)** HTHS versus LTLS condition. Red, blue, green, and black text indicates upregulation, downregulation, both up and downregulation, and no changes of pathways or (proteins. LHCs, light harvesting complexes; PSII, photosystem II; PSI, photosystem I; PPDK, pyruvate phosphate kinase; PEPC, phosphoenolpyruvate; CA, carbonic anhydrase; PRK, phosphoribulokinase; rbcL, Rubisco large subunit; rbcS, Rubisco small subunit; PGK, phosphoglycerate kinase; TKT2, fructose-bisphosphate aldolase; GAPDH, glyceraldehyde-3-phosphate dehydrogenase; pgm, phosphoglucomutase; GPI, glucose-6-phosphate isomerase; FEP, fructose-1,6-bisphosphatase; pfka, 6-phosphofructokinase; ALDO, fructose-bisphosphate aldolase; GAPD, glyceraldehyde-3-phosphate dehydrogenase; TPI, triose-phosphate isomerase; ENO, α-enolase; PYK, pyruvate kinase; G6PD, glugose-6-phospahate 1-dehydrogenase; PGLS, 6-phosphogluconolactonase; PGD, 6-phosphogluconate dehrdrogenase; talA, transaldolase; LAT2, pyruvate dehydrogenase E2 (dihydrolipoamide *s*-acetyltransferase); PDHA1, pyruvate dehydrogenase E1 component subunit alpha-1, PDHB1, pyruvate dehydrogenase E1 component subunit beta-1; ACACA, acetyl-CoA carboxylase; ACSL, long chan acyl-COA synthetases, CS, citrate synthase; ACO, aconitate hydratase 2; icd, isocitrate dehydrogenase; SDHA, succinate dehydrogenase; gcvT, glycine decarboxylase T protein; gcvH, glycine decarboxylase H protein; gcvP, glycine decarboxylase P protein; SHMT, glycine/serine hydroxymethyltransferase; AGXT, alanine-glyoxylate transaminase; GLN, glutamine synthetase; NIR_2, Ferredoxin0nitrite reductase; URE, urease; NRT, nitrate/nitrite transporters; SIT, silicic acid transporter. The comprehensive information of all the identified regulated proteins associated with carbon metabolism are given in Supplementary Tables S2, S3. These images were created using Corel Draw Graphics suite.)

The combining elevation of temperature and silicate limitation affect the photosynthetic efficiency of *S. dohrnii*, as shown in Figure 6. As marine diatom fixes 20% of global CO_2_, their photosynthetic efficiency enhanced by RuBisCO as it involves in the first carbon step of carbon fixation. In this study, RuBisCO was downregulated by both temperature and silicate limitation (Supplementary Table S2). Further, many proteins involved in the Calvin cycle were down-regulated in HTHS versus LTLS (Figure 7) shows combining temperature or silicate stress suppressed C3 photosynthesis and carbon fixation, and influence in the carbon that inside the cell toward RuBisCO are adjusted to the match reductant supply (Allen et al., 2008).

Diatom has major enzymes of phosphoenolpyruvate carboxylase (PEPC), phosphoenolpyruvate carboxykinase, and pyruvate phosphate dikinase (PPDK) to perform C4 photosynthesis (Armbrust et al., 2004; Poulson-Ellestad et al., 2014). In this study PEPC was differentially expressed in HTHS versus HTLS and HTHS versus LTHS condition, whereas, the PPDK was only differentially expressed in HTHS versus LTLS (Figure 7), indicating suppression of C4 photosynthesis by both temperature and silicate stress. The regulated PPDK and carbonic anhydrase in our study show the proteomic modification of *S. dohrnii* central carbon metabolism (CCM). Similarly, many downregulating genes related to CCM were observed in the nutrient-limited condition of the diatom (Alipanah et al., 2015). In this study, most of the glycolysis and TCA proteins were upregulated under HTHS versus HTLS cells, while most of the proteins were decreased in HTHS versus LTHS cells (Figure 7), indicating that these metabolisms were sensitive to the temperature changes. Among them, increased abundance of pyruvate dehydrogenase (PDHA) in HTHS versus HTLS, HTHS versus LTHS, and HTHS versus LTLS shows direct carbon away from intracellular carbohydrate store to the TCA cycle (Figure 7). The TCA cycle in diatom generally upregulated in response to high levels of amino acids, and protein degradation, which creates intermediates and provides for nutrient assimilation bender (Lyon et al., 2011). Further, under combining temperature and silicate stress of HTHS versus LTLS, citrate synthase, and succinate dehydrogenase (SDH) were also regulated (Figure 7), which catalyzes succinate–fumarate coupling and then direct to the oxidation of TCA cycle, that intermediate with the photosynthetic electron chain. The up-regulation of (G3P) protein in HTHS versus HTLS shows *S. dohrnii* would enhance the Calvin cycle by catalyzing the dephosphorylation of 1,3-bisphosphoglycerateacid (BPGA) to produce glyceraldehyde-3-phosphate (GAP) (Trost et al., 2006). Down-regulation of phosphoglycerate kinase (PDHA1) in HTLS versus LTHS (See Supplementary Table S2) indicating reduced efficiency step 7 process of glycolysis. However, it shows the opposite trend as upregulation proteins in HTHS versus HTLS, and HTHS versus LTLS, (Figure 7) resulting an increasing process. Collectively, these gene expressions indicating that combine temperature and silicate stress reduced these processes in diatom *S. dohrnii.*

Metabolic regulation of glycolytic proteins shows that nutrient deficiency has a different bypass mechanism in diatom (Dyhrman et al., 2012). Similarly, the glycolysis, and the pentose phosphate pathway generates NAD(P)H and pentoses (5-carbon sugars) as well as ribose 5-phosphate. Collectively, both stress condition (temperature and silicate) activated the phosphate-reaction participation (Figure 7) and enhance the capacity of NAD(P)H production, aiming to promote silicate utilization under lower silicate condition and supply under reduction of ATP. It is clear that when combining temperature and silicate changes occur in the environment, there are positive and negative impacts on diatom carbon metabolism; regulation of RuBisCO would alter the efficiency of diatom carbon fixation. Our findings are in line with previous studies experimental demonstrations in response to the limitation of CO_2_, silicate, and ocean acidification of plankton and oysters (Allen et al., 2008; Clement et al., 2017; Jian et al., 2017).

### Downregulation of Ribosome Biogenesis

Ribosome biogenesis is vital to a cellular process for all the living organisms, but remarkably insufficient information is known about the underlying pathway. Compared to prokaryotes (i.e., yeasts) much is poorly known about the ribosome biogenesis pathway in plants, animals and particularly of marine planktons (Henras et al., 2008). Thomson et al. (2013) reported that the core of higher eukaryotic ribosome biogenesis pathway was different from the yeast counterpart. Based on the KEGG pathway analysis, eight ribosome biogenesis proteins were observed during the changes of temperature and silicate (HTLS) from these six proteins were downregulated (green color) (Supplementary Table S4 and Figure S6) including three 90S-pre-ribosome components (CK2A, CK2B, UTP6), these proteins play an essential coenzyme and enzyme regulator in ribosome biogenesis pathway.

Furthermore, it is interacting selectively and non-covalently with small nucleolar RNA and non-covalently with ATP, adenosine 5′-triphosphate. The other three rRNA modification proteins NKP6, DKC1, and KRE33, which involved in catalysis of the reactions (RNA uridine = RNA pseudouridine). It converts the uridine in RNA molecule to pseudouridine by rotation of the C1′-N-1 glycosidic bond of uridine in RNA to a C1′-C5 (Foster et al., 2000). The protein KRE33 also involved in catalysis of the reaction: acetyl-CoA + cytidine = CoA + N4-acetylcytidine. The precise role of these downregulated proteins is yet to be defined. Two proteins were upregulated (red color) in ribosome biogenesis SNU13 and Ran which involved in nucleocytoplasmic transport and chromatin condensation and control of cell cycle. However, there is also evidence for differences in the ribosome biogenesis pathway between higher temperature and lower temperature, and lower silicate level during the culture of diatoms *S. dohrnii*. In our proteomic profiling, based on the temperature (HTLS vs. LTHS) four proteins were upregulating (i.e., CK2A, CK2B, NOP56, KRE33), which intricate in the metabolic functions and support the RNA involving in the processing of ribosomes and reduce the oxidoreductase activity (Smith et al., 2013). Three proteins were downregulating (SNU13, EMG1, Ran), which modulates the activity of protein kinase and involved in chromatin condensation in the cell cycle. SNU13 protein is involving in the maturation of a precursor Large SubUnit (LSU) ribosomal RNA (rRNA) molecule into a mature LSU-rRNA molecule (Ebersberger et al., 2014). Besides, in our diatom, proteomic profiling of LTHS versus LTLS, five genes (Imp3, NOP58, NOP56, Nog1, CRM1) were downregulating with lower temperature and silicate (Supplementary Table S2). The Imp3 gene involved in the conversion of an important ribosomal RNA (rRNA) transcript into one or more mature rRNA molecules and protein NOP58, NOP56 involves the DNA binding and snoRNA binding in cells. Besides, one gene NAN1 was upregulating in this study, NAN1 serves as a metal-binding cofactor and bound tightly inactive sites or loosely with the substrate. In our study, the downregulated proteins Imp3, NOP58, NOP56, Nog1, CRM1 be a specific response to combining changes of temperature and limitation of silicate could affect the cell growth and cell cycle of marine diatom *S. dohrnii.*

### Protein Biomarkers

The PCA and PLS-DA model applied to discriminate the results of diatom *S. dohrnii* iTRAQ data. PCA and PLS-DA score plots demonstrated the good separation between higher temperature, lower temperature versus higher silicate and lower silicate level of culture conditions. The higher value of VIP score indicates the great contribution of the proteins to the discrimination of diatom samples in this study. Fifteen protein biomarkers were identified based on the VIP scores given in Table 1. Two proteins (EJK63051.1) and (EJK50298.1) play a vital role in the regulation of metabolic process including cell cycle, growth, DNA binding, and transmitting the signals throughout the cell. Gene, atpB encodes a subunit of mitochondrial ATP synthase, it catalyses ATP synthesis utilizing an electrochemical gradient of protons across the inner membrane during oxidative phosphorylation (Weber, 2006). The other protein, (AIE44732.1) or gene rbcL plays a critical role in carbon dioxide fixation, as well as the oxidative fragmentation of the pentose substrate in the photorespiration process (Chen et al., 2018). Furthermore, two proteins (EJK69871.1) and (EED92818.1) involved in the regulation of actin cytoskeleton organization and endocytosis (Cope et al., 1999).

### Gene Redundancy

The different biochemical pathways were identified from different diatoms including photosynthesis (Grouneva et al., 2011), and carbon cycle in *S. costatum* (Zhang et al., 2015), urea cycle in *T. pseudonana* (Armbrust et al., 2004), and mitochondrial in *P. tricornutum* (Kroth et al., 2008). Additionally, the phylogenetic affinities to other organisms such as the genes of bacterial (Bowler et al., 2008), and green algal origins identified in *T. pseudonana* and *P. tricornutum* (Moustafa et al., 2009). The cellular metabolic pathways and processes influenced by the different stress condition in *S. dohrnii* are given in Figure 7. In KEGG pathways, Genes, PDHA1, PDHB1 catalyzes the overall conversion of pyruvate to acetyl-CoA and CO(2) and provides the primary link between glycolysis and the tricarboxylic acid (TCA) cycle. Similar to this study, Carvalho and Lettieri (2011) were reported that redundant gene (THAPSDRAFT_25042) in *T. pseudonana* responsible for the upregulation of metabolic process (long-chain acyl-CoA synthetase), upon exposure to benzo(a)pyrene. The following photosystem PSI and II two encoded proteins or genes were identified as part of the photosynthetic electron transport of diatom *T. pseudonana* (Grouneva et al., 2011) in *P. tricornutum* (Alipanah et al., 2015) in *S. costatum* (Zhang et al., 2015). PS1 subunits consists of PsaA, PsaB, PsaL, PsaE, PsaJ, PsaM, and PSII units consists of PsbO (O), PsbU, PsbV, Psb31, PsbQ. In our study, we demonstrated the response of these genes on photosynthesis in different stress condition and these results were confirmed in diatom *S. dohrnii*. Additionally, Carvalho and Lettieri (2011) were reported the upregulated proteins or gene, cfxX (RuBisCO expression protein) and Lhcr4, Lhcr10 (fucoxanthin chl a/c light-harvesting protein) responsible for the regulation of photosynthesis in *T. pseudonana* upon exposure to benzo(a)pyrene. The genes (PPdk, PGK, pfkA, PYK1, PDHA1, and PDHB1) responsible for the regulation of carbon metabolism of model diatom *P. tricornutum* during N deprivation (Alipanah et al., 2015), were also reported in field-collected and laboratory-cultured *S. costatum* (Zhang et al., 2015). In our study, the regulation of carbon metabolism was assessed in *S. dohrnii* and confirmed the involvement of above-mentioned genes in carbon fixation process.

## Conclusion

Our investigation of the responses of diatom *S. dohrnii* to the concomitant warming ocean and limiting nutrients (Si) with multivariate statistics and pathway analysis gave new insights into intracellular metabolic processes. We observed downregulation of the photosynthesis process, carbon assimilation and utilization mechanisms and biological processes related to cell cycle regulation during elevated temperature and Si limitation in the diatom. Further protein biomarkers revealed signal transduction, ATP production, and carbon fixation pathways associated to thermal and nutrient stress. Our findings contribute to our understanding of the impact of climate change on the physiological adjustment to molecular mechanism of diatoms.

## Data Availability Statement

The raw proteomics data and analysis files for the manuscript have been submitted to ProteomeXchange via Pride database (www.ebi.ac.uk/pride/archive/) with identifier PXD022918.

## Author Contributions

ST and JS designed this study. ST performed the laboratory experiment, carried out data analysis, and defined the manuscript contents in discussion with SP and JS. GZ performed pigment analysis and drafted pigment part. JS coordinated this investigation and provided guidance and facilities to perform this experiment. All authors contributed to the article and approved the submitted version.

## Conflict of Interest

The authors declare that the research was conducted in the absence of any commercial or financial relationships that could be construed as a potential conflict of interest.
