# Supplementary material for: Quantitative Proteomic Profiling of Marine Diatom Skeletonema dohrnii in Response to Temperature and Silicate Induced Environmental Stress
Source: Front Microbiol. 2021 Jan 14;11:554832. doi: 10.3389/fmicb.2020.554832 (PMC7841394; doi:10.3389/fmicb.2020.554832)
Supplement: Supplementary file 4 [file Data_Sheet_4.doc]

**Title: Quantitative proteomic profiling of marine diatom *Skeletonema dohrnii* in response to temperature and silicate induced environmental stress**

**Authors:** Satheeswaran Thangaraj, Satheesh Kumar Palanisamy, Guicheng Zhang, Jun Sun*

**Supplementary Table S4:** General chemical methods, Figures and Tables

**General methods**

**Chemical composition used for the preparation of ASW**

The used composition of anhydrous salts NaCl (24.54g); (Na_2_SO_4_, 4.09 g); (KCL, 0.7 g); (NaHCO_3_, 0.2 g), (KBr, 0.1 g), (H_3_BO_3_, 0.003 g), (NaF, 0.003 g), and the Hydrous Salts (MgCl_2_,11.10 g), (CaCl_2_, 1.54 g), (SrCl_2_, 0.017 g) were dissolved in 950 mL of de-ionize water and brought the volume to 1 L after adding nutrients, trace metal and vitamin described below.

Major nutrients were prepared as a stock solution (NaH_2_PO_4_, 1.38 g L-1), (NaNO_3_, 8.50 gL-1) and (Na_2_SiO_3_, 28.40 g L-1) and 1 mL of each solution was added to 1 L of medium. For the metal stock solution, first (CuSO_4_, 4.9 g L-1) and (Na_2_SeO_3_, 1.9 g L-1) were prepared as individual stock solution, then chemicals (EDTA 29.2 g), (FeCl_3_, 0.27 g), (ZnSO_4_, 0.023g), (MnCl_2_, 0.0024 g), (CoCl_2_, 0.012 g), (Na_2_MoO_4_, 0.024 g), (CuSO_4_, 4.9 g) and (Na2SeO_3_, 1.9g) were dissolved in 950 mL of de-ionized water, then 1 mL of each stock solution were added and brought the volume to 1 L finally. For the medium 1 L of final solution was added to the 1 L of medium. To prepare the vitamin stock solution initially cyanocobalamin (5.5gL-1) and Biotin (5.0g L-1) were prepared as an individual solution. Then to prepare the mixed vitamin solution in 950 mL, cyanocobalamin stock solution 1 mL, Biotin stock solution 1 mL and Thiamine (100 mg) was added and brought to 1L. For each liter of ASW medium 1 ml of stock, vitamin stock solution was used. All the chemicals were used in the culture medium were purchased from Sigma – Aldrich (USA).

| 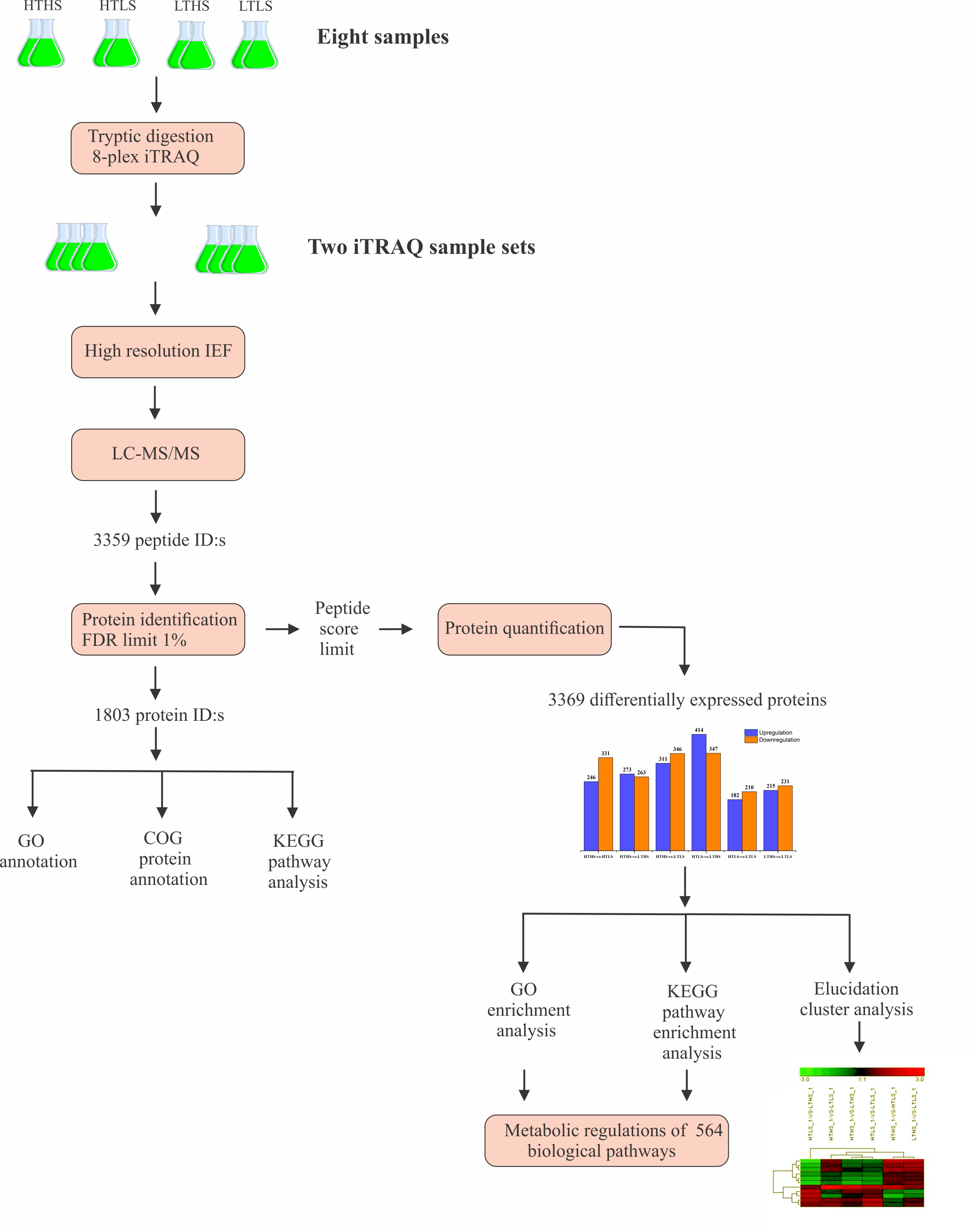 |
| --- |
| **Fig. S1**. Experimental setup and carried out bioinformatics analysis of this study. |


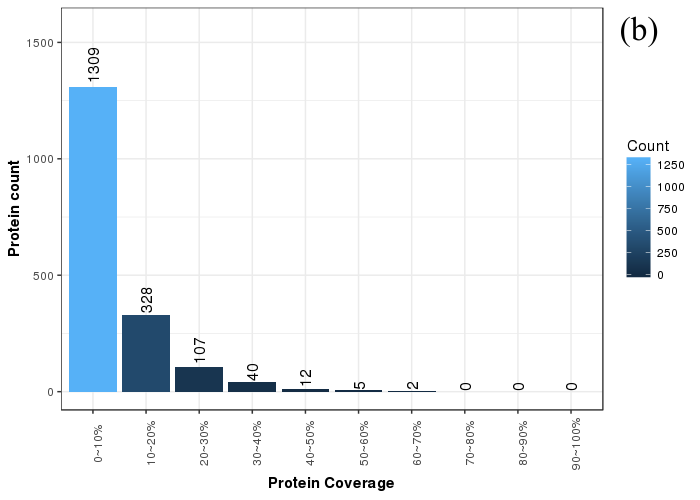

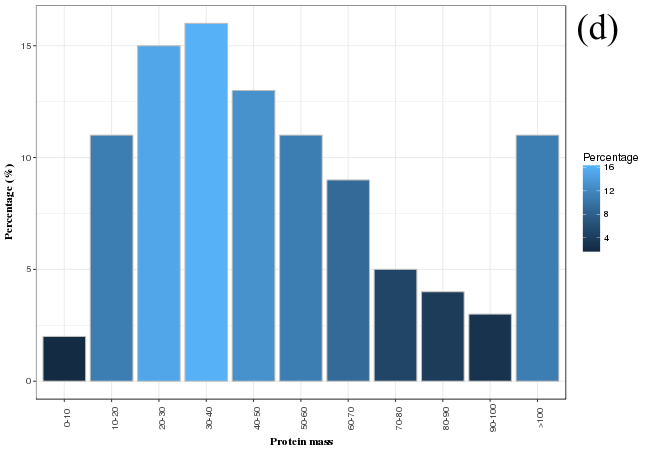


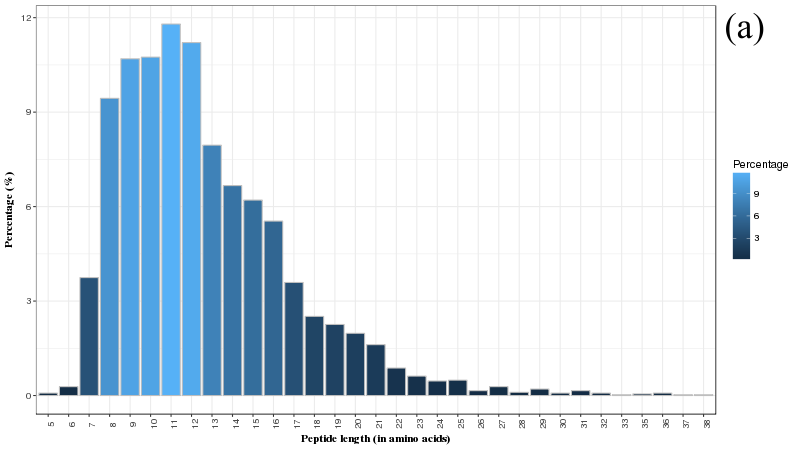


(c)

**
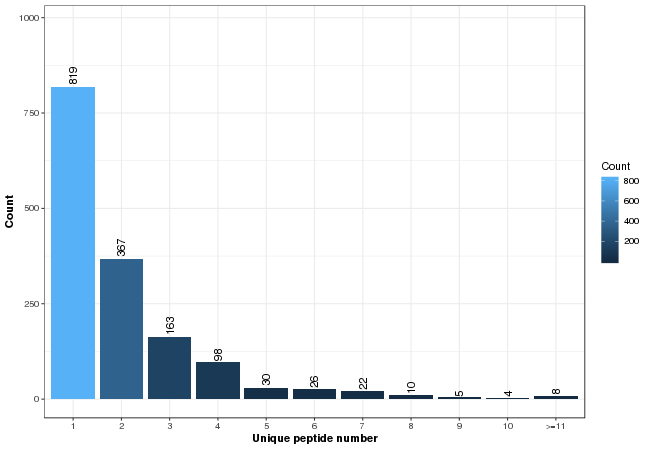
**


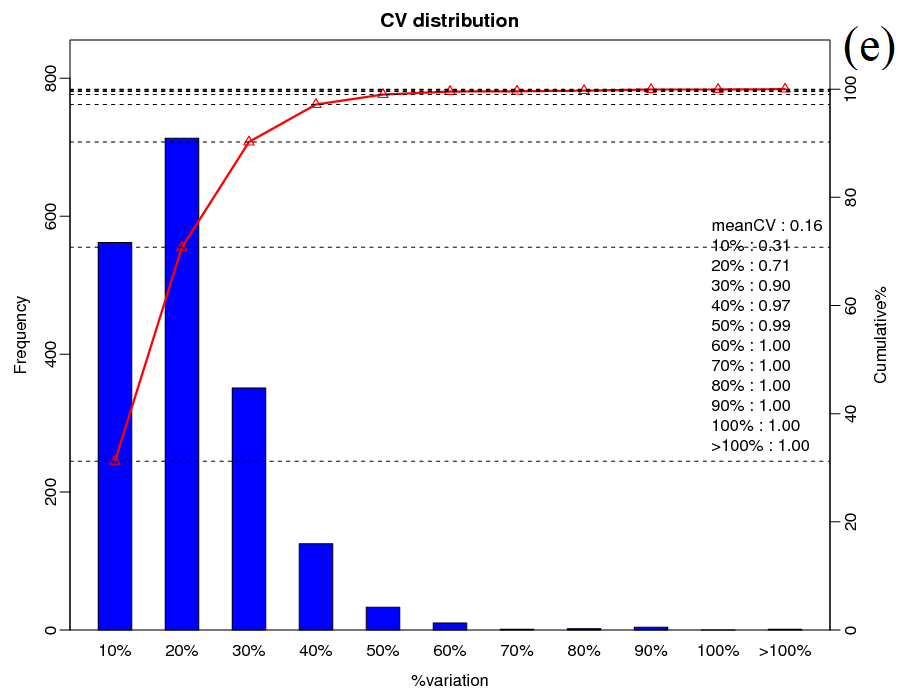


**Fig. S2.** Peptide and protein statistics. The distributions of peptide length (a), protein spectrum coverage (b), unique peptide number distribution identified using Mascot version 2.3.02 against selected database (FDR of 1%) (c), protein mass distribution (d) protein coverage were determined by iTRAQ analysis in the entire study (e) cv distribution of peptide determined by iTRAQ analysis.


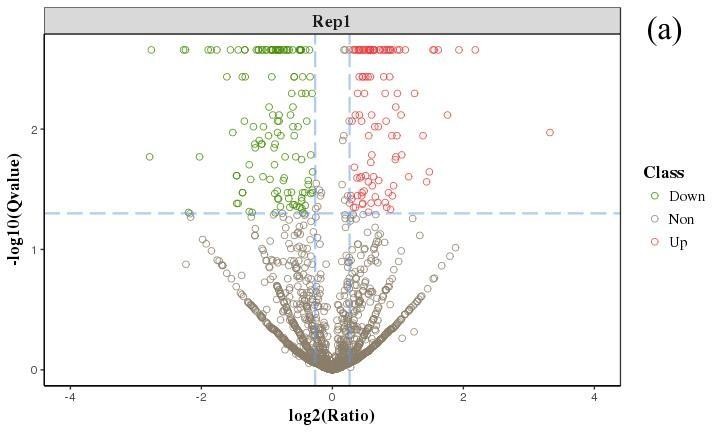

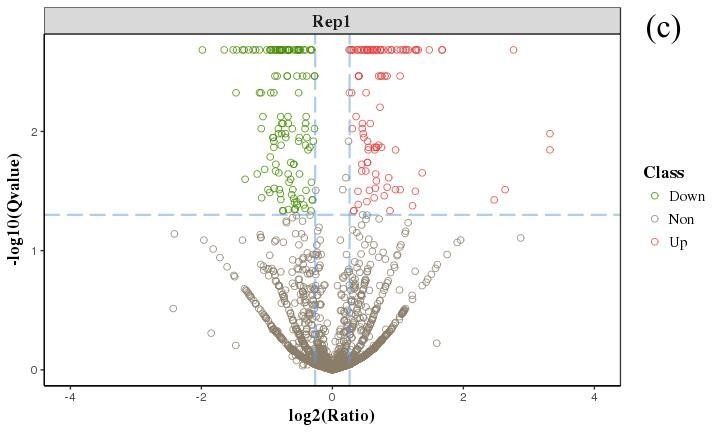

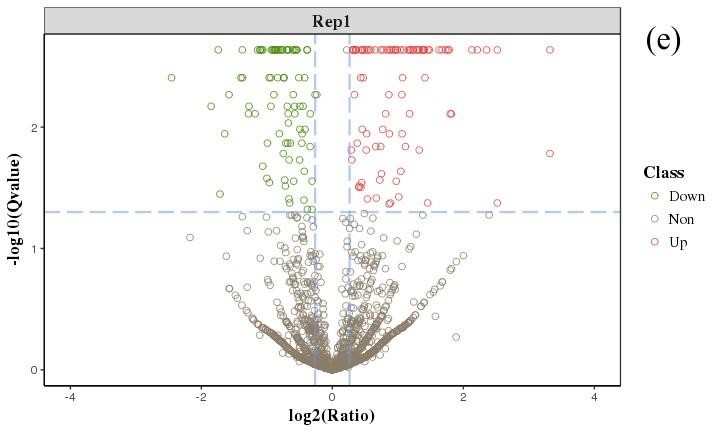

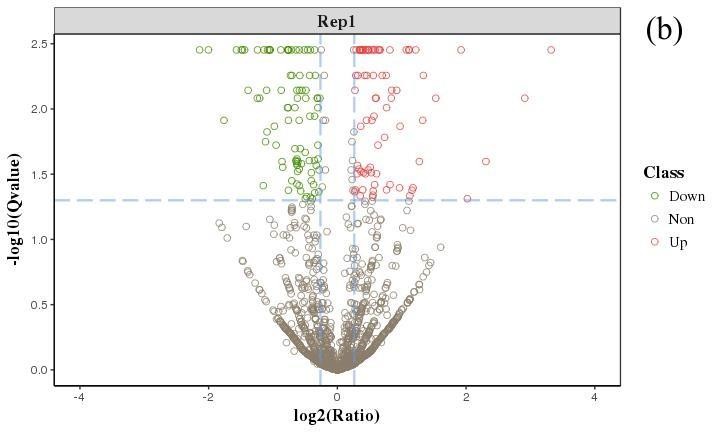

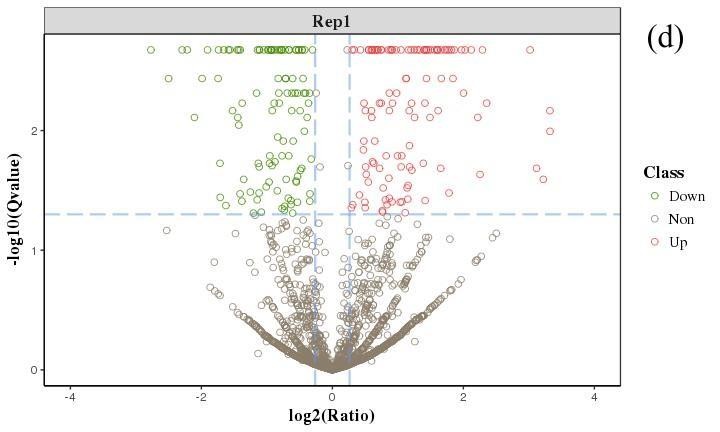

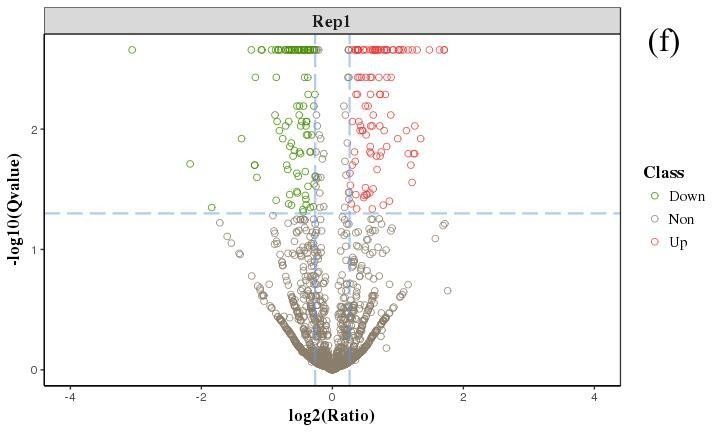


**Fig. S3**. Identified differently expressed proteins by volcano plot analysis. Differentially expressed protein of HTHS vs. HTLS (a), HTHS vs. LTHS (b), HTHS vs. LTLS (c), HTLS vs. LTHS (d), LTHS vs. LTLS (e), LTHS vs.

LTLS (f) This plot depicts volcano plot of log2 fold-change (x-axis) versus -log10 Q-value (y-axis, representing the probability that the protein is differentially expressed). Q-value <0.05 and Foldchange > 1.2 are set as the significant threshold for differentially expression. The red and green dots indicate points-of-interest that display both large- magnitude fold-changes as well as high statistical significance. Dots in red mean significant up-regulated proteins which passed screening threshold. Dots in green mean significant down-regulated proteins which passed screening threshold. And grey dots are non-significant differentially expressed protein.

| 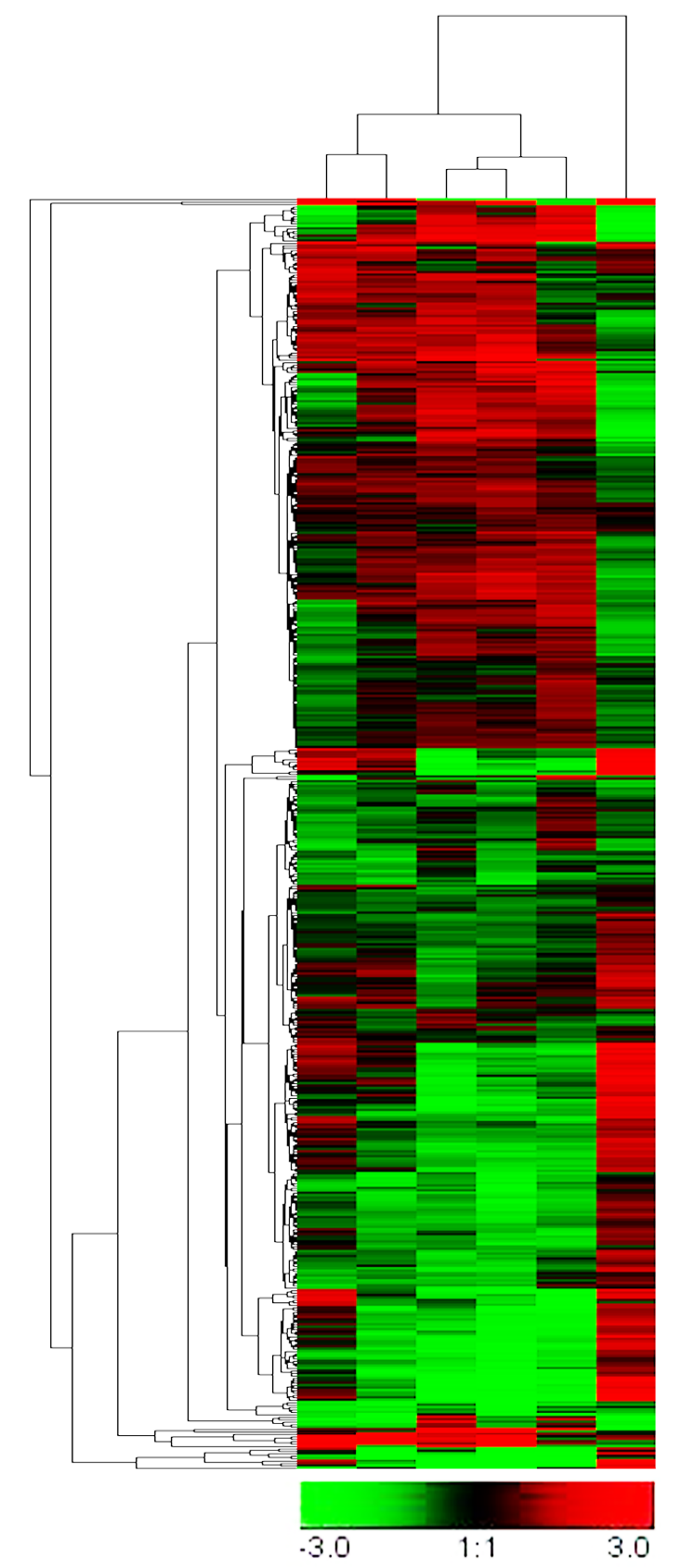 |
| --- |
| **Fig. S4.** Cluster analysis of whole gene expression profiles. Green indicates down-regulation, red indicates upregulation, Grey indicates no detectable expression. Array (1) HTHS vs. HTLS; (2) HTHS vs. LTHS; (3) HTHS vs. LTLS; (4) HTLS vs. LTHS; (5) HTLS vs. LTLS (6) LTHS vs. LTLS |

| 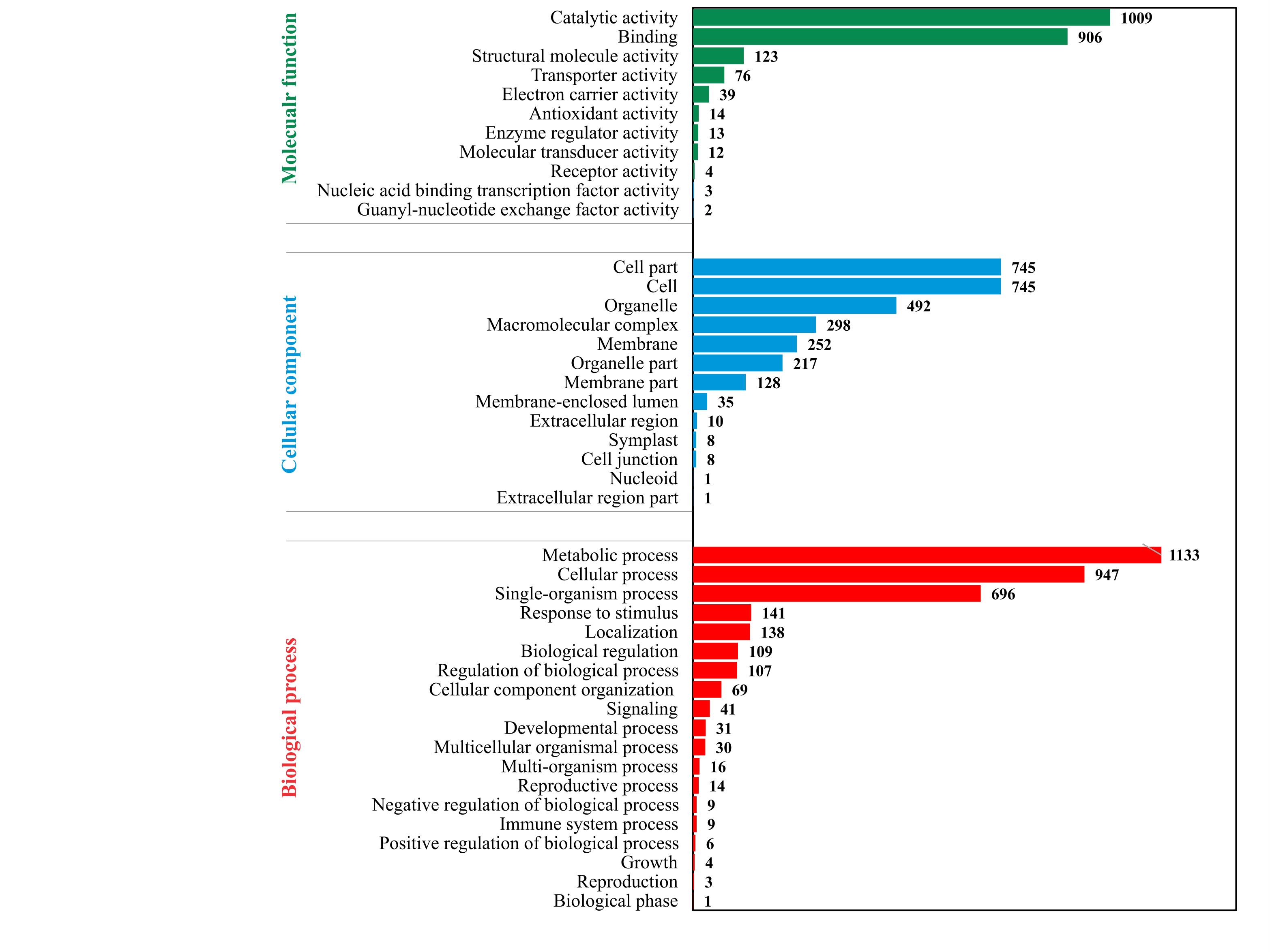 |
| --- |
| **Fig. S5.** Bar plot of the Gene Ontology (GO) analysis. The bar chart shows the distribution of corresponding GO terms. Different colors represent different GO categories. The GO analyzes were done for differentially expressed proteins before KEGG pathway analysis that were found around all conditions in this study. |

| 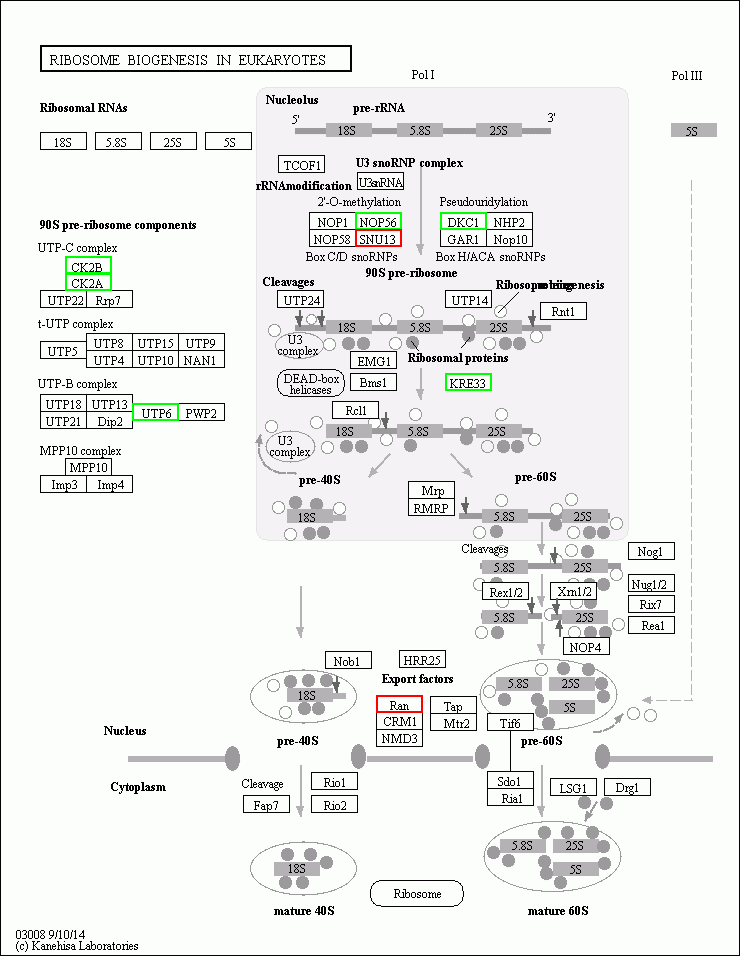 |
| --- |
| **Fig. S6**. The ribosome biogenesis pathway map for *Skeletonema dohrnii* during the changes of temperature and silicate (HTHS vs. HTLS). The down-regulated proteins were shown in light green color boxes and up- regulated proteins are given in red color**.** |

| **Table S4A**. Number of unique proteins family of other species relevant to *S. dohrnii* species. | |
| --- | --- |
| **Species name** | The number of unique protein family |
| *Thalassiosira pseudonana* | 1030 |
| *Thalassiosira oceanica* | 672 |
| Cyclotella sp. | 21 |
| *Thalassiosira weissflogii* | 21 |
| *Roundia cardiophora* | 20 |
| *Skeletonema costatum* | 12 |
| *Skeletonema marinoi* | 5 |
| *Cyclotella cryptica* | 4 |
| *Detonula confervacea* | 3 |
| *Detonula confervacea* | 3 |
| *Thalassiosira nordenskioeldii* | 2 |
| Thalassiosira sp | 2 |
| *Thalassiosira nordenskioeldii* | 2 |
| *Cyclotella meneghiniana* | 2 |
| *Stephanodiscus hantzschii* | 1 |
| *Cyclotella striata* | 1 |
| *Skeletonema tropicum* | 1 |
| *Thalassiosira punctigera* | 1 |
| *Skeletonema pseudocostatum* | 1 |

| **Table S4B**. Significantly enriched KEGG pathways in *S. dohrnii* under **HTHS vs. HTLS** condition. | | | |
| --- | --- | --- | --- |
| Si | KEGG Pathway | Differential expressed protein number | *P*-value |
| 1 | Ribosome | 68 | 5.54E-11 |
| 2 | Photosynthesis | 24 | 0.000841 |
| 3 | Photosynthesis - antenna proteins | 13 | 0.007265 |
| 4 | Oxidative phosphorylation | 24 | 0.019885 |
| 5 | Phagosome | 11 | 0.07924 |
| 6 | Riboflavin metabolism | 4 | 0.085863 |
| 7 | Spliceosome | 16 | 0.094507 |
| 8 | Monoterpenoid biosynthesis | 2 | 0.101409 |
| 9 | Steroid biosynthesis | 2 | 0.101409 |
| 10 | Endocytosis | 13 | 0.123436 |
| 11 | Arachidonic acid metabolism | 4 | 0.151002 |
| 12 | ABC transporters | 4 | 0.228806 |
| 13 | Thiamine metabolism | 4 | 0.228806 |
| 14 | Other types of O-glycan biosynthesis | 2 | 0.239782 |
| 15 | mRNA surveillance pathway | 9 | 0.241989 |
| 16 | Nicotinate and nicotinamide metabolism | 5 | 0.252502 |
| 17 | RNA transport | 14 | 0.266667 |
| 18 | Protein export | 6 | 0.267998 |
| 19 | Purine metabolism | 25 | 0.279071 |
| 20 | MAPK signalling pathway - plant | 4 | 0.313745 |
| 21 | Nucleotide excision repair | 4 | 0.313745 |
| 22 | Biotin metabolism | 4 | 0.313745 |
| 23 | Indole alkaloid biosynthesis | 1 | 0.318681 |
| 24 | AGE-RAGE signalling pathway in diabetic complications | 1 | 0.318681 |
| 25 | Butanoate metabolism | 2 | 0.381345 |
| 26 | Base excision repair | 2 | 0.381345 |
| 27 | Glycerophospholipid metabolism | 4 | 0.400651 |
| 28 | Carbon fixation in photosynthetic organisms | 19 | 0.418089 |
| 29 | Pyrimidine metabolism | 16 | 0.427218 |
| 30 | Fatty acid biosynthesis | 8 | 0.458858 |
| 31 | Ribosome biogenesis in eukaryotes | 8 | 0.458858 |
| 32 | RNA degradation | 11 | 0.493643 |
| 33 | Circadian rhythm – plant | 2 | 0.510039 |
| 34 | Glycolysis / Gluconeogenesis | 20 | 0.522475 |
| 35 | N-Glycan biosynthesis | 1 | 0.535954 |
| 36 | Fatty acid metabolism | 9 | 0.559266 |
| 37 | RNA polymerase | 5 | 0.614662 |
| 38 | Homologous recombination | 2 | 0.619686 |
| 39 | Inositol phosphate metabolism | 4 | 0.636749 |

| 40 | Fructose and mannose metabolism | 5 | 0.674832 |
| --- | --- | --- | --- |
| 41 | Plant hormone signal transduction | 1 | 0.68404 |
| 42 | Protein processing in endoplasmic reticulum | 13 | 0.686158 |
| 43 | Glycine, serine and threonine metabolism | 12 | 0.697546 |
| 44 | Mismatch repair | 2 | 0.70934 |
| 45 | Histidine metabolism | 2 | 0.70934 |
| 46 | Glycerolipid metabolism | 2 | 0.70934 |
| 47 | Aminoacyl-tRNA biosynthesis | 14 | 0.709845 |
| 48 | Phenylalanine, tyrosine and tryptophan biosynthesis | 5 | 0.728418 |
| 49 | Fatty acid degradation | 3 | 0.734021 |
| 50 | DNA replication | 3 | 0.734021 |
| 51 | One carbon pool by folate | 3 | 0.734021 |
| 52 | Pyruvate metabolism | 14 | 0.74182 |
| 53 | Sulfur metabolism | 4 | 0.755931 |
| 54 | Arginine and proline metabolism | 5 | 0.775323 |
| 55 | Terpenoid backbone biosynthesis | 2 | 0.780586 |
| 56 | Galactose metabolism | 3 | 0.836594 |
| 57 | Pentose and glucuronate interconversions | 3 | 0.836594 |
| 58 | Valine, leucine and isoleucine degradation | 4 | 0.842463 |
| 59 | alpha-Linolenic acid metabolism | 1 | 0.853664 |
| 60 | Amino sugar and nucleotide sugar metabolism | 6 | 0.858375 |
| 61 | Porphyrin and chlorophyll metabolism | 6 | 0.858375 |
| 62 | Glutathione metabolism | 7 | 0.866607 |
| 63 | Plant-pathogen interaction | 2 | 0.878492 |
| 64 | Phosphatidylinositol signalling system | 1 | 0.900459 |
| 65 | Tyrosine metabolism | 1 | 0.900459 |
| 66 | Propanoate metabolism | 5 | 0.903049 |
| 67 | Selenocompound metabolism | 2 | 0.910597 |
| 68 | Glyoxylate and dicarboxylate metabolism | 12 | 0.917508 |
| 69 | Carbon metabolism | 42 | 0.926523 |
| 70 | Citrate cycle (TCA cycle) | 8 | 0.92896 |
| 71 | Tropane, piperidine and pyridine alkaloid biosynthesis | 1 | 0.932312 |
| 72 | Biosynthesis of unsaturated fatty acids | 1 | 0.932312 |
| 73 | Vitamin B6 metabolism | 1 | 0.932312 |
| 74 | Alanine, aspartate and glutamate metabolism | 8 | 0.941845 |
| 75 | Metabolic pathways | 167 | 0.949483 |
| 76 | beta-Alanine metabolism | 1 | 0.953987 |
| 77 | Ascorbate and aldarate metabolism | 2 | 0.96557 |
| 78 | Peroxisome | 3 | 0.968567 |
| 79 | Monobactam biosynthesis | 1 | 0.968732 |
| 80 | Nitrogen metabolism | 2 | 0.975177 |
| 81 | Lysine biosynthesis | 1 | 0.985574 |
| 82 | Proteasome | 6 | 0.991215 |

| 83 | Carotenoid biosynthesis | 1 | 0.993354 |
| --- | --- | --- | --- |
| 84 | Ubiquitin mediated proteolysis | 2 | 0.995398 |
| 85 | Arginine biosynthesis | 1 | 0.999048 |
| 86 | Valine, leucine and isoleucine biosynthesis | 1 | 0.999048 |
| 87 | Pentose phosphate pathway | 2 | 0.999437 |
| 88 | Cysteine and methionine metabolism | 4 | 0.999883 |
| 89 | Biosynthesis of secondary metabolites | 70 | 0.999894 |
| 90 | Biosynthesis of amino acids | 27 | 0.999963 |
| 91 | 2-Oxocarboxylic acid metabolism | 1 | 0.999987 |

| **Table S4C.** The significantly enriched KEGG pathways in *S. dohrnii* under **HTHS vs. LTHS** condition. | | | |
| --- | --- | --- | --- |
| Si | KEGG Pathway | Differential expressed protein number | *P*-value |
| 1 | Ribosome | 45 | 0.010963 |
| 2 | Photosynthesis | 20 | 0.015161 |
| 3 | Propanoate metabolism | 11 | 0.053342 |
| 4 | Inositol phosphate metabolism | 7 | 0.061665 |
| 5 | Phosphatidylinositol signalling system | 4 | 0.070169 |
| 6 | One carbon pool by folate | 6 | 0.0776 |
| 7 | Glyoxylate and dicarboxylate metabolism | 20 | 0.081449 |
| 8 | Carbon fixation in photosynthetic organisms | 22 | 0.083614 |
| 9 | Biotin metabolism | 5 | 0.098302 |
| 10 | Fatty acid metabolism | 12 | 0.100969 |
| 11 | Pyruvate metabolism | 19 | 0.115872 |
| 12 | Fatty acid biosynthesis | 10 | 0.118542 |
| 13 | Nitrogen metabolism | 7 | 0.130226 |
| 14 | Glycerophospholipid metabolism | 5 | 0.149735 |
| 15 | Aminoacyl-tRNA biosynthesis | 18 | 0.160694 |
| 16 | Carbon metabolism | 52 | 0.177602 |
| 17 | Photosynthesis - antenna proteins | 9 | 0.185172 |
| 18 | Metabolic pathways | 177 | 0.22901 |
| 19 | Riboflavin metabolism | 3 | 0.255626 |
| 20 | Phenylpropanoid biosynthesis | 1 | 0.300137 |
| 21 | Indole alkaloid biosynthesis | 1 | 0.300137 |
| 22 | Caffeine metabolism | 1 | 0.300137 |
| 23 | Alanine, aspartate and glutamate metabolism | 13 | 0.30039 |
| 24 | Biosynthesis of unsaturated fatty acids | 3 | 0.353086 |
| 25 | Vitamin B6 metabolism | 3 | 0.353086 |
| 26 | Glutathione metabolism | 10 | 0.363685 |
| 27 | Pentose phosphate pathway | 9 | 0.372442 |
| 28 | Citrate cycle (TCA cycle) | 12 | 0.390738 |
| 29 | Glycolysis / Gluconeogenesis | 20 | 0.394194 |
| 30 | Valine, leucine and isoleucine degradation | 6 | 0.403426 |

| 31 | Protein export | 5 | 0.416136 |
| --- | --- | --- | --- |
| 32 | Ascorbate and aldarate metabolism | 5 | 0.416136 |
| 33 | Fatty acid degradation | 4 | 0.430842 |
| 34 | beta-Alanine metabolism | 3 | 0.448663 |
| 35 | Arginine biosynthesis | 6 | 0.466266 |
| 36 | alpha-Linolenic acid metabolism | 2 | 0.472205 |
| 37 | N-Glycan biosynthesis | 1 | 0.510337 |
| 38 | Folate biosynthesis | 1 | 0.510337 |
| 39 | Monoterpenoid biosynthesis | 1 | 0.510337 |
| 40 | Sulfur relay system | 1 | 0.510337 |
| 41 | Peroxisome | 6 | 0.527198 |
| 42 | Arginine and proline metabolism | 6 | 0.527198 |
| 43 | Purine metabolism | 21 | 0.547798 |
| 44 | Ribosome biogenesis in eukaryotes | 7 | 0.561493 |
| 45 | RNA transport | 11 | 0.578106 |
| 46 | Homologous recombination | 2 | 0.580494 |
| 47 | Protein processing in endoplasmic reticulum | 13 | 0.585232 |
| 48 | Other types of O-glycan biosynthesis | 1 | 0.657505 |
| 49 | Tryptophan metabolism | 1 | 0.657505 |
| 50 | Cyan amino acid metabolism | 1 | 0.657505 |
| 51 | Arachidonic acid metabolism | 2 | 0.671457 |
| 52 | Histidine metabolism | 2 | 0.671457 |
| 53 | Biosynthesis of amino acids | 42 | 0.687738 |
| 54 | Selenocompound metabolism | 3 | 0.688499 |
| 55 | DNA replication | 3 | 0.688499 |
| 56 | Nicotinate and nicotinamide metabolism | 3 | 0.688499 |
| 57 | mRNA surveillance pathway | 6 | 0.688714 |
| 58 | Pyrimidine metabolism | 13 | 0.692866 |
| 59 | RNA degradation | 9 | 0.698718 |
| 60 | Phagosome | 6 | 0.733616 |
| 61 | Oxidative phosphorylation | 14 | 0.738071 |
| 62 | Biosynthesis of secondary metabolites | 86 | 0.738479 |
| 63 | ABC transporters | 2 | 0.745722 |
| 64 | Terpenoid backbone biosynthesis | 2 | 0.745722 |
| 65 | RNA polymerase | 4 | 0.755928 |
| 66 | Butanoate metabolism | 1 | 0.760513 |
| 67 | Base excision repair | 1 | 0.760513 |
| 68 | Cysteine and methionine metabolism | 11 | 0.760564 |
| 69 | Nucleotide excision repair | 2 | 0.805098 |
| 70 | Spliceosome | 9 | 0.827471 |
| 71 | Phenylalanine, tyrosine and tryptophan biosynthesis | 4 | 0.837381 |
| 72 | Endocytosis | 7 | 0.843526 |
| 73 | Starch and sucrose metabolism | 1 | 0.883009 |

| 74 | Ubiquinone and other terpenoid-quinone biosynthesis | 1 | 0.883009 |
| --- | --- | --- | --- |
| 75 | Lysine biosynthesis | 2 | 0.888108 |
| 76 | Glycine, serine and threonine metabolism | 9 | 0.909179 |
| 77 | Amino sugar and nucleotide sugar metabolism | 5 | 0.911655 |
| 78 | Mismatch repair | 1 | 0.918268 |
| 79 | Carotenoid biosynthesis | 2 | 0.937283 |
| 80 | Thiamine metabolism | 1 | 0.942917 |
| 81 | Proteasome | 7 | 0.958141 |
| 82 | Pantothenate and CoA biosynthesis | 1 | 0.960145 |
| 83 | MAPK signalling pathway - plant | 1 | 0.960145 |
| 84 | Monobactam biosynthesis | 1 | 0.960145 |
| 85 | Sulfur metabolism | 2 | 0.965485 |
| 86 | Ubiquitin mediated proteolysis | 3 | 0.965522 |
| 87 | Porphyrin and chlorophyll metabolism | 4 | 0.96796 |
| 88 | Plant-pathogen interaction | 1 | 0.972181 |
| 89 | Fructose and mannose metabolism | 2 | 0.981281 |
| 90 | 2-Oxocarboxylic acid metabolism | 1 | 0.999972 |

| **Table S4D.** The significantly enriched KEGG pathways in *S. dohrnii* under **HTHS vs. LTLS** condition. | | | |
| --- | --- | --- | --- |
| Si | KEGG Pathway | Differential expressed protein number | *P*-value |
| 1 | Ribosome | 64 | 5.30E-06 |
| 2 | Carbon fixation in photosynthetic organisms | 32 | 0.001424 |
| 3 | Glyoxylate and dicarboxylate metabolism | 28 | 0.004227 |
| 4 | Riboflavin metabolism | 5 | 0.028584 |
| 5 | Carbon metabolism | 68 | 0.038368 |
| 6 | Propanoate metabolism | 13 | 0.043466 |
| 7 | Arginine and proline metabolism | 11 | 0.050894 |
| 8 | Aminoacyl-tRNA biosynthesis | 23 | 0.076645 |
| 9 | Pyruvate metabolism | 23 | 0.095848 |
| 10 | Proteasome | 18 | 0.096127 |
| 11 | Plant-pathogen interaction | 6 | 0.119975 |
| 12 | beta-Alanine metabolism | 5 | 0.130364 |
| 13 | N-Glycan biosynthesis | 2 | 0.136882 |
| 14 | Monoterpenoid biosynthesis | 2 | 0.136882 |
| 15 | Glutathione metabolism | 14 | 0.141794 |
| 16 | Glycolysis / Gluconeogenesis | 27 | 0.169852 |
| 17 | Fatty acid biosynthesis | 11 | 0.192567 |
| 18 | Ascorbate and aldarate metabolism | 7 | 0.229037 |
| 19 | Arachidonic acid metabolism | 4 | 0.233947 |
| 20 | Glycerolipid metabolism | 4 | 0.233947 |
| 21 | Protein processing in endoplasmic reticulum | 19 | 0.239726 |

| 22 | Valine, leucine and isoleucine degradation | 8 | 0.267106 |
| --- | --- | --- | --- |
| 23 | Glycerophospholipid metabolism | 5 | 0.293838 |
| 24 | Other types of O-glycan biosynthesis | 2 | 0.309538 |
| 25 | Biosynthesis of secondary metabolites | 115 | 0.321129 |
| 26 | Fatty acid metabolism | 12 | 0.322519 |
| 27 | Inositol phosphate metabolism | 6 | 0.338824 |
| 28 | Pyrimidine metabolism | 19 | 0.363668 |
| 29 | Phenylpropanoid biosynthesis | 1 | 0.370192 |
| 30 | Indole alkaloid biosynthesis | 1 | 0.370192 |
| 31 | AGE-RAGE signalling pathway in diabetic complications | 1 | 0.370192 |
| 32 | Caffeine metabolism | 1 | 0.370192 |
| 33 | RNA polymerase | 7 | 0.374714 |
| 34 | Selenocompound metabolism | 5 | 0.385218 |
| 35 | One carbon pool by folate | 5 | 0.385218 |
| 36 | RNA transport | 15 | 0.386082 |
| 37 | Phosphatidylinositol signalling system | 3 | 0.393958 |
| 38 | Pantothenate and CoA biosynthesis | 4 | 0.442027 |
| 39 | MAPK signalling pathway - plant | 4 | 0.442027 |
| 40 | Biotin metabolism | 4 | 0.442027 |
| 41 | Fructose and mannose metabolism | 7 | 0.450039 |
| 42 | Citrate cycle (TCA cycle) | 14 | 0.470165 |
| 43 | Base excision repair | 2 | 0.472807 |
| 44 | Ribosome biogenesis in eukaryotes | 9 | 0.495068 |
| 45 | Mismatch repair | 3 | 0.513967 |
| 46 | Alanine, aspartate and glutamate metabolism | 14 | 0.521345 |
| 47 | Steroid biosynthesis | 1 | 0.603503 |
| 48 | mRNA surveillance pathway | 8 | 0.605622 |
| 49 | alpha-Linolenic acid metabolism | 2 | 0.609988 |
| 50 | Purine metabolism | 25 | 0.636645 |
| 51 | Protein export | 5 | 0.639194 |
| 52 | Phagosome | 8 | 0.664584 |
| 53 | 2-Oxocarboxylic acid metabolism | 10 | 0.679426 |
| 54 | Glycine, serine and threonine metabolism | 14 | 0.705459 |
| 55 | Nitrogen metabolism | 5 | 0.707839 |
| 56 | Monobactam biosynthesis | 3 | 0.70873 |
| 57 | Nucleotide excision repair | 3 | 0.70873 |
| 58 | Arginine biosynthesis | 6 | 0.710496 |
| 59 | Homologous recombination | 2 | 0.718004 |
| 60 | Ubiquinone and other terpenoid-quinone biosynthesis | 2 | 0.718004 |
| 61 | RNA degradation | 11 | 0.730882 |
| 62 | Cysteine and methionine metabolism | 14 | 0.744036 |
| 63 | Cyanoamino acid metabolism | 1 | 0.750485 |
| 64 | Metabolic pathways | 204 | 0.761674 |

| 65 | Peroxisome | 6 | 0.764838 |
| --- | --- | --- | --- |
| 66 | Carotenoid biosynthesis | 4 | 0.771352 |
| 67 | Pentose and glucuronate interconversions | 4 | 0.771352 |
| 68 | Spliceosome | 12 | 0.773617 |
| 69 | Tropane, piperidine and pyridine alkaloid biosynthesis | 2 | 0.799618 |
| 70 | Biosynthesis of unsaturated fatty acids | 2 | 0.799618 |
| 71 | Vitamin B6 metabolism | 2 | 0.799618 |
| 72 | Pentose phosphate pathway | 8 | 0.806721 |
| 73 | DNA replication | 3 | 0.837301 |
| 74 | Nicotinate and nicotinamide metabolism | 3 | 0.837301 |
| 75 | Lysine biosynthesis | 3 | 0.837301 |
| 76 | Endocytosis | 9 | 0.840255 |
| 77 | Butanoate metabolism | 1 | 0.843044 |
| 78 | Isoquinoline alkaloid biosynthesis | 1 | 0.843044 |
| 79 | Valine, leucine and isoleucine biosynthesis | 5 | 0.856832 |
| 80 | Biosynthesis of amino acids | 49 | 0.85705 |
| 81 | ABC transporters | 2 | 0.859548 |
| 82 | Terpenoid backbone biosynthesis | 2 | 0.859548 |
| 83 | Sulfur metabolism | 4 | 0.866533 |
| 84 | Porphyrin and chlorophyll metabolism | 7 | 0.876673 |
| 85 | Galactose metabolism | 3 | 0.913668 |
| 86 | Phenylalanine metabolism | 1 | 0.937969 |
| 87 | Tyrosine metabolism | 1 | 0.937969 |
| 88 | Fatty acid degradation | 2 | 0.95442 |
| 89 | Ubiquitin mediated proteolysis | 4 | 0.970952 |
| 90 | Thiamine metabolism | 1 | 0.975524 |
| 92 | Photosynthesis | 10 | 0.982955 |
| 93 | Phenylalanine, tyrosine and tryptophan biosynthesis | 3 | 0.984815 |
| 94 | Photosynthesis - antenna proteins | 2 | 0.999505 |
| 95 | Oxidative phosphorylation | 9 | 0.999551 |
| 96 | Amino sugar and nucleotide sugar metabolism | 1 | 0.999992 |

| **Table S4E.** The significantly enriched KEGG pathways in *S. dohrnii* under **HTLS vs. LTHS** condition. | | | |
| --- | --- | --- | --- |
| Si | KEGG Pathway | Differential expressed protein number | *P*-value |
| 1 | Photosynthesis - antenna proteins | 19 | 2.82E-05 |
| 2 | Ribosome | 61 | 0.006436 |
| 3 | Selenocompound metabolism | 9 | 0.009806 |
| 4 | Vitamin B6 metabolism | 6 | 0.026916 |
| 5 | Photosynthesis | 25 | 0.028752 |
| 6 | Glycine, serine and threonine metabolism | 24 | 0.028856 |

| 7 | Biosynthesis of amino acids | 73 | 0.046166 |
| --- | --- | --- | --- |
| 8 | Nitrogen metabolism | 10 | 0.053647 |
| 9 | Fructose and mannose metabolism | 10 | 0.136488 |
| 10 | One carbon pool by folate | 7 | 0.136694 |
| 11 | Glycolysis / Gluconeogenesis | 31 | 0.148841 |
| 12 | Endocytosis | 16 | 0.160293 |
| 13 | Oxidative phosphorylation | 26 | 0.176609 |
| 14 | N-Glycan biosynthesis | 2 | 0.182917 |
| 15 | Steroid biosynthesis | 2 | 0.182917 |
| 16 | Alanine, aspartate and glutamate metabolism | 19 | 0.184263 |
| 17 | Phenylalanine, tyrosine and tryptophan biosynthesis | 10 | 0.193842 |
| 18 | Thiamine metabolism | 5 | 0.219188 |
| 19 | Amino sugar and nucleotide sugar metabolism | 13 | 0.230128 |
| 20 | Phagosome | 12 | 0.239352 |
| 21 | Sulfur metabolism | 8 | 0.283057 |
| 22 | Inositol phosphate metabolism | 7 | 0.29627 |
| 23 | Galactose metabolism | 7 | 0.29627 |
| 24 | Lysine biosynthesis | 6 | 0.310777 |
| 25 | Histidine metabolism | 4 | 0.345182 |
| 26 | Glycerolipid metabolism | 4 | 0.345182 |
| 27 | Arginine biosynthesis | 9 | 0.347972 |
| 28 | Ascorbate and aldarate metabolism | 7 | 0.387 |
| 29 | Plant hormone signal transduction | 2 | 0.392504 |
| 30 | Cyanoamino acid metabolism | 2 | 0.392504 |
| 31 | Peroxisome | 9 | 0.427458 |
| 32 | Phenylpropanoid biosynthesis | 1 | 0.427885 |
| 33 | AGE-RAGE signalling pathway in diabetic complications | 1 | 0.427885 |
| 34 | Caffeine metabolism | 1 | 0.427885 |
| 35 | Glycerophospholipid metabolism | 5 | 0.437996 |
| 36 | RNA degradation | 15 | 0.443162 |
| 37 | mRNA surveillance pathway | 10 | 0.480962 |
| 38 | Aminoacyl-tRNA biosynthesis | 21 | 0.501861 |
| 39 | Pentose and glucuronate interconversions | 6 | 0.508463 |
| 40 | Phosphatidylinositol signalling system | 3 | 0.513446 |
| 41 | Riboflavin metabolism | 3 | 0.513446 |
| 42 | Protein processing in endoplasmic reticulum | 19 | 0.537434 |
| 43 | Spliceosome | 16 | 0.541304 |
| 44 | Fatty acid degradation | 5 | 0.544013 |
| 45 | DNA replication | 5 | 0.544013 |
| 46 | Fatty acid biosynthesis | 10 | 0.553528 |
| 47 | Cysteine and methionine metabolism | 18 | 0.556351 |
| 48 | Base excision repair | 2 | 0.572521 |
| 49 | MAPK signalling pathway - plant | 4 | 0.586591 |

| 50 | Monobactam biosynthesis | 4 | 0.586591 |
| --- | --- | --- | --- |
| 51 | Nucleotide excision repair | 4 | 0.586591 |
| 52 | Metabolic pathways | 241 | 0.590449 |
| 53 | Pentose phosphate pathway | 11 | 0.594961 |
| 54 | Protein export | 6 | 0.59956 |
| 55 | Mismatch repair | 3 | 0.639644 |
| 56 | Arachidonic acid metabolism | 3 | 0.639644 |
| 57 | Carbon fixation in photosynthetic organisms | 23 | 0.654276 |
| 58 | Monoterpenoid biosynthesis | 1 | 0.672852 |
| 59 | Sulfur relay system | 1 | 0.672852 |
| 60 | Carbon metabolism | 64 | 0.685099 |
| 61 | Circadian rhythm - plant | 2 | 0.70989 |
| 62 | Fatty acid metabolism | 11 | 0.713533 |
| 63 | beta-Alanine metabolism | 3 | 0.740724 |
| 64 | Nicotinate and nicotinamide metabolism | 4 | 0.766592 |
| 65 | RNA transport | 14 | 0.782618 |
| 66 | Purine metabolism | 27 | 0.803157 |
| 67 | Homologous recombination | 2 | 0.808111 |
| 68 | Starch and sucrose metabolism | 2 | 0.808111 |
| 69 | Ubiquinone and other terpenoid-quinone biosynthesis | 2 | 0.808111 |
| 70 | Tyrosine metabolism | 2 | 0.808111 |
| 71 | Lysine degradation | 1 | 0.813026 |
| 72 | Other types of O-glycan biosynthesis | 1 | 0.813026 |
| 73 | Tryptophan metabolism | 1 | 0.813026 |
| 74 | Biotin metabolism | 3 | 0.817791 |
| 75 | Ribosome biogenesis in eukaryotes | 8 | 0.839956 |
| 76 | Biosynthesis of secondary metabolites | 121 | 0.848535 |
| 77 | Glutathione metabolism | 10 | 0.865477 |
| 78 | Biosynthesis of unsaturated fatty acids | 2 | 0.875498 |
| 79 | Butanoate metabolism | 1 | 0.893195 |
| 80 | Valine, leucine and isoleucine degradation | 5 | 0.916914 |
| 81 | Ubiquitin mediated proteolysis | 6 | 0.920387 |
| 82 | ABC transporters | 2 | 0.920423 |
| 83 | Terpenoid backbone biosynthesis | 2 | 0.920423 |
| 84 | Glyoxylate and dicarboxylate metabolism | 17 | 0.924059 |
| 85 | Propanoate metabolism | 7 | 0.924421 |
| 86 | alpha-Linolenic acid metabolism | 1 | 0.939021 |
| 87 | Pantothenate and CoA biosynthesis | 2 | 0.949746 |
| 88 | Pyrimidine metabolism | 15 | 0.955417 |
| 89 | RNA polymerase | 4 | 0.959407 |
| 90 | Phenylalanine metabolism | 1 | 0.965203 |
| 92 | Proteasome | 11 | 0.96577 |
| 93 | Plant-pathogen interaction | 2 | 0.968577 |
| 94 | Citrate cycle (TCA cycle) | 10 | 0.980037 |

| 95 | Tropane, piperidine and pyridine alkaloid biosynthesis | 1 | 0.980154 |
| --- | --- | --- | --- |
| 96 | Porphyrin and chlorophyll metabolism | 6 | 0.985501 |
| 97 | Arginine and proline metabolism | 4 | 0.987713 |
| 98 | Carotenoid biosynthesis | 2 | 0.992659 |
| 99 | Pyruvate metabolism | 13 | 0.994563 |
| 100 | 2-Oxocarboxylic acid metabolism | 5 | 0.999226 |
| 101 | Valine, leucine and isoleucine biosynthesis | 2 | 0.999413 |

| **Table S4F.** The significantly enriched KEGG pathways in *S. dohrnii* under **HTLS vs. LTLS** condition. | | | |
| --- | --- | --- | --- |
| Si | KEGG Pathway | Differential expressed protein number | *P*-value |
| 1 | Thiamine metabolism | 5 | 0.013642 |
| 2 | Vitamin B6 metabolism | 4 | 0.041357 |
| 3 | Selenocompound metabolism | 5 | 0.063992 |
| 4 | One carbon pool by folate | 5 | 0.063992 |
| 5 | Carbon metabolism | 41 | 0.064985 |
| 6 | beta-Alanine metabolism | 4 | 0.069073 |
| 7 | Pyruvate metabolism | 15 | 0.080114 |
| 8 | Inositol phosphate metabolism | 5 | 0.123234 |
| 9 | Protein processing in endoplasmic reticulum | 13 | 0.124972 |
| 10 | Arachidonic acid metabolism | 3 | 0.171745 |
| 11 | Carbon fixation in photosynthetic organisms | 15 | 0.196866 |
| 12 | Base excision repair | 2 | 0.201833 |
| 13 | Ether lipid metabolism | 1 | 0.213599 |
| 14 | Phenylpropanoid biosynthesis | 1 | 0.213599 |
| 15 | Caffeine metabolism | 1 | 0.213599 |
| 16 | Metabolic pathways | 127 | 0.239452 |
| 17 | Ubiquitin mediated proteolysis | 6 | 0.24127 |
| 18 | Proteasome | 10 | 0.251477 |
| 19 | Cysteine and methionine metabolism | 11 | 0.272153 |
| 20 | alpha-Linolenic acid metabolism | 2 | 0.290679 |
| 21 | Pyrimidine metabolism | 12 | 0.290691 |
| 22 | Pantothenate and CoA biosynthesis | 3 | 0.298015 |
| 23 | Nucleotide excision repair | 3 | 0.298015 |
| 24 | Endocytosis | 8 | 0.300763 |
| 25 | Photosynthesis - antenna proteins | 6 | 0.323082 |
| 26 | Protein export | 4 | 0.348983 |
| 27 | Ascorbate and aldarate metabolism | 4 | 0.348983 |
| 28 | Propanoate metabolism | 6 | 0.365448 |
| 29 | Glycine, serine and threonine metabolism | 10 | 0.374995 |
| 30 | Glyoxylate and dicarboxylate metabolism | 12 | 0.37588 |
| 31 | Homologous recombination | 2 | 0.378086 |

| 32 | Phosphatidylinositol signalling system | 2 | 0.378086 |
| --- | --- | --- | --- |
| 33 | N-Glycan biosynthesis | 1 | 0.381689 |
| 34 | Monoterpenoid biosynthesis | 1 | 0.381689 |
| 35 | Steroid biosynthesis | 1 | 0.381689 |
| 36 | Sulfur relay system | 1 | 0.381689 |
| 37 | Arginine and proline metabolism | 5 | 0.382869 |
| 38 | RNA transport | 9 | 0.390896 |
| 39 | Nitrogen metabolism | 4 | 0.403142 |
| 40 | Fatty acid degradation | 3 | 0.427842 |
| 41 | DNA replication | 3 | 0.427842 |
| 42 | Mismatch repair | 2 | 0.460623 |
| 43 | Biosynthesis of unsaturated fatty acids | 2 | 0.460623 |
| 44 | Glycerolipid metabolism | 2 | 0.460623 |
| 45 | Valine, leucine and isoleucine degradation | 4 | 0.508008 |
| 46 | Cyanoamino acid metabolism | 1 | 0.513941 |
| 47 | Citrate cycle (TCA cycle) | 8 | 0.515914 |
| 48 | ABC transporters | 2 | 0.536381 |
| 49 | Purine metabolism | 15 | 0.543496 |
| 50 | Galactose metabolism | 3 | 0.54821 |
| 51 | Valine, leucine and isoleucine biosynthesis | 4 | 0.55729 |
| 52 | Ribosome biogenesis in eukaryotes | 5 | 0.564999 |
| 53 | Biosynthesis of amino acids | 31 | 0.569458 |
| 54 | Fatty acid metabolism | 6 | 0.571846 |
| 55 | Glycolysis / Gluconeogenesis | 13 | 0.582547 |
| 56 | Photosynthesis | 9 | 0.589258 |
| 57 | Monobactam biosynthesis | 2 | 0.604486 |
| 58 | Glutathione metabolism | 6 | 0.609353 |
| 59 | Butanoate metabolism | 1 | 0.617977 |
| 60 | Glycerophospholipid metabolism | 2 | 0.664742 |
| 61 | Plant-pathogen interaction | 2 | 0.664742 |
| 62 | Pentose phosphate pathway | 5 | 0.681741 |
| 63 | RNA polymerase | 3 | 0.697622 |
| 64 | Circadian rhythm - plant | 1 | 0.699802 |
| 65 | Oxidative phosphorylation | 10 | 0.702401 |
| 66 | Spliceosome | 7 | 0.70655 |
| 67 | Alanine, aspartate and glutamate metabolism | 7 | 0.70655 |
| 68 | Biosynthesis of secondary metabolites | 61 | 0.712175 |
| 69 | Lysine biosynthesis | 2 | 0.717387 |
| 70 | mRNA surveillance pathway | 4 | 0.724251 |
| 71 | Aminoacyl-tRNA biosynthesis | 9 | 0.728098 |
| 72 | Fructose and mannose metabolism | 3 | 0.738254 |
| 73 | RNA degradation | 6 | 0.739439 |
| 74 | Fatty acid biosynthesis | 4 | 0.757777 |
| 75 | Starch and sucrose metabolism | 1 | 0.764145 |
| 76 | Phenylalanine metabolism | 1 | 0.764145 |

| 77 | Riboflavin metabolism | 1 | 0.764145 |
| --- | --- | --- | --- |
| 78 | Arginine biosynthesis | 3 | 0.774446 |
| 79 | Carotenoid biosynthesis | 2 | 0.801949 |
| 80 | Pentose and glucuronate interconversions | 2 | 0.801949 |
| 81 | Peroxisome | 3 | 0.806442 |
| 82 | Histidine metabolism | 1 | 0.814732 |
| 83 | Terpenoid backbone biosynthesis | 1 | 0.854496 |
| 84 | Sulfur metabolism | 2 | 0.863304 |
| 85 | Biotin metabolism | 1 | 0.885747 |
| 86 | 2-Oxocarboxylic acid metabolism | 4 | 0.896882 |
| 87 | Ribosome | 19 | 0.906124 |
| 88 | Amino sugar and nucleotide sugar metabolism | 3 | 0.928075 |
| 89 | Porphyrin and chlorophyll metabolism | 3 | 0.928075 |
| 90 | Nicotinate and nicotinamide metabolism | 1 | 0.929595 |
| 92 | Phagosome | 2 | 0.972041 |
| 93 | Phenylalanine, tyrosine and tryptophan biosynthesis | 1 | 0.987145 |

| **Table S4G.** The significantly enriched KEGG pathways in *S. dohrnii* under **LTHS vs. LTLS** condition. | | | |
| --- | --- | --- | --- |
| Si | KEGG Pathway | Differential expressed protein number | *P*-value |
| 1 | Biosynthesis of amino acids | 50 | 0.01522984 |
| 2 | Glycine, serine and threonine metabolism | 17 | 0.02151314 |
| 3 | Sulfur metabolism | 8 | 0.021816 |
| 4 | Cysteine and methionine metabolism | 17 | 0.02770129 |
| 5 | Fructose and mannose metabolism | 8 | 0.04989129 |
| 6 | Biosynthesis of secondary metabolites | 90 | 0.05029148 |
| 7 | Glutathione metabolism | 12 | 0.05102062 |
| 8 | Pentose phosphate pathway | 11 | 0.05174627 |
| 9 | Carbon metabolism | 49 | 0.06157619 |
| 10 | Glycerolipid metabolism | 4 | 0.08064983 |
| 11 | Ribosome | 36 | 0.08206747 |
| 12 | Amino sugar and nucleotide sugar metabolism | 10 | 0.08941657 |
| 13 | Metabolic pathways | 158 | 0.1221634 |
| 14 | beta-Alanine metabolism | 4 | 0.1289806 |
| 15 | Protein export | 6 | 0.1309382 |
| 16 | Lysine biosynthesis | 5 | 0.1322085 |
| 17 | Glycolysis / Gluconeogenesis | 20 | 0.1629882 |
| 18 | Tryptophan metabolism | 2 | 0.1686008 |
| 19 | Pantothenate and CoA biosynthesis | 4 | 0.1862299 |
| 20 | MAPK signalling pathway - plant | 4 | 0.1862299 |
| 21 | Biotin metabolism | 4 | 0.1862299 |
| 22 | Pyrimidine metabolism | 15 | 0.2221943 |

| 23 | Inositol phosphate metabolism | 5 | 0.2336765 |
| --- | --- | --- | --- |
| 24 | Galactose metabolism | 5 | 0.2336765 |
| 25 | Glycerophospholipid metabolism | 4 | 0.2497897 |
| 26 | Plant-pathogen interaction | 4 | 0.2497897 |
| 27 | Ether lipid metabolism | 1 | 0.260989 |
| 28 | AGE-RAGE signalling pathway in diabetic complications | 1 | 0.260989 |
| 29 | Arachidonic acid metabolism | 3 | 0.2664093 |
| 30 | Selenocompound metabolism | 4 | 0.3169789 |
| 31 | Porphyrin and chlorophyll metabolism | 8 | 0.3172228 |
| 32 | 2-Oxocarboxylic acid metabolism | 9 | 0.3355505 |
| 33 | Nitrogen metabolism | 5 | 0.3498805 |
| 34 | Protein processing in endoplasmic reticulum | 13 | 0.35319 |
| 35 | Proteasome | 11 | 0.3649973 |
| 36 | Arginine and proline metabolism | 6 | 0.3738695 |
| 37 | RNA polymerase | 5 | 0.4095262 |
| 38 | Monobactam biosynthesis | 3 | 0.4301848 |
| 39 | Photosynthesis | 12 | 0.4506278 |
| 40 | Folate biosynthesis | 1 | 0.4539953 |
| 41 | Monoterpenoid biosynthesis | 1 | 0.4539953 |
| 42 | Steroid biosynthesis | 1 | 0.4539953 |
| 43 | Starch and sucrose metabolism | 2 | 0.492185 |
| 44 | Phosphatidylinositol signaling system | 2 | 0.492185 |
| 45 | Riboflavin metabolism | 2 | 0.492185 |
| 46 | Ascorbate and aldarate metabolism | 4 | 0.5175124 |
| 47 | Fatty acid degradation | 3 | 0.5789413 |
| 48 | One carbon pool by folate | 3 | 0.5789413 |
| 49 | Fatty acid biosynthesis | 6 | 0.5806795 |
| 50 | Ribosome biogenesis in eukaryotes | 6 | 0.5806795 |
| 51 | Phagosome | 6 | 0.5806795 |
| 52 | Mismatch repair | 2 | 0.5824953 |
| 53 | Plant hormone signal transduction | 1 | 0.5966926 |
| 54 | Lysine degradation | 1 | 0.5966926 |
| 55 | Cyanoamino acid metabolism | 1 | 0.5966926 |
| 56 | Fatty acid metabolism | 7 | 0.6254085 |
| 57 | Purine metabolism | 17 | 0.68353 |
| 58 | Valine, leucine and isoleucine degradation | 4 | 0.6859962 |
| 59 | Pentose and glucuronate interconversions | 3 | 0.7007069 |
| 60 | Butanoate metabolism | 1 | 0.7021687 |
| 61 | Base excision repair | 1 | 0.7021687 |
| 62 | Endocytosis | 7 | 0.7034746 |
| 63 | Nucleotide excision repair | 2 | 0.7261391 |
| 64 | Arginine biosynthesis | 4 | 0.7319339 |
| 65 | Phenylalanine, tyrosine and tryptophan biosynthesis | 4 | 0.7319339 |

| 66 | Valine, leucine and isoleucine biosynthesis | 4 | 0.7319339 |
| --- | --- | --- | --- |
| 67 | Carbon fixation in photosynthetic organisms | 13 | 0.7397546 |
| 68 | Propanoate metabolism | 5 | 0.7577272 |
| 69 | Peroxisome | 4 | 0.7726452 |
| 70 | alpha-Linolenic acid metabolism | 1 | 0.7801135 |
| 71 | Circadian rhythm - plant | 1 | 0.7801135 |
| 72 | Alanine, aspartate and glutamate metabolism | 8 | 0.7903172 |
| 73 | RNA degradation | 7 | 0.7989246 |
| 74 | DNA replication | 2 | 0.8256571 |
| 75 | Homologous recombination | 1 | 0.8376992 |
| 76 | Tyrosine metabolism | 1 | 0.8376992 |
| 77 | mRNA surveillance pathway | 4 | 0.8660277 |
| 78 | Tropane, piperidine and pyridine alkaloid biosynthesis | 1 | 0.8802332 |
| 79 | Histidine metabolism | 1 | 0.8802332 |
| 80 | Vitamin B6 metabolism | 1 | 0.8802332 |
| 81 | Terpenoid backbone biosynthesis | 1 | 0.911642 |
| 82 | Thiamine metabolism | 1 | 0.911642 |
| 83 | Ubiquitin mediated proteolysis | 3 | 0.926414 |
| 84 | Glyoxylate and dicarboxylate metabolism | 9 | 0.9368517 |
| 85 | Citrate cycle (TCA cycle) | 6 | 0.9388992 |
| 86 | Photosynthesis - antenna proteins | 3 | 0.9527527 |
| 87 | Aminoacyl-tRNA biosynthesis | 8 | 0.9590746 |
| 88 | Nicotinate and nicotinamide metabolism | 1 | 0.9645731 |
| 89 | Pyruvate metabolism | 8 | 0.9654138 |
| 90 | Carotenoid biosynthesis | 1 | 0.9807607 |
| 92 | RNA transport | 5 | 0.9809134 |
| 93 | Oxidative phosphorylation | 7 | 0.9921469 |

| **Table S4H. Metabolic regulation of photosynthetic pigment proteins between each conditions** | | | |
| --- | --- | --- | --- |
| **HTHS vs HTLS** | | | |
| No | Protein ID | Description | Fold change |
| 1 | EJK46744.1 | Chlorophyll a-b binding protein 1 | -1.4 |
| 2 | CAI44219.1 | fucoxanthin chlorophyll a/c binding protein | -1.4 |
| 3 | EJK73893.1 | Fucoxanthin-chlorophyll a-c binding protein F | -1.1 |
| 4 | AAB70098.1 | fucoxanthin-chlorophyll a/c light-harvesting protein | -1.1 |
| 5 | XP_002294116.1 | Fucoxanthin-chlorophyll a-c binding protein F | -1.0 |
| 6 | XP_002297311.1 | Photosystem I light harvesting protein | -1.0 |
| 7 | EED95211.1 | fucoxanthin chlorophyll a/c protein | -0.9 |
| 8 | EED93059.1 | Fucoxanthin-chlorophyll a-c binding protein A | -0.7 |
| 9 | EED87666.1 | Fucoxanthin-chlorophyll a-c binding protein | -0.6 |
| 11 | EJK58980.1 | Fucoxanthin-chlorophyll a-c binding protein E | -0.6 |
| 12 | CAA04403.1 | Fucoxanthin-chlorophyll a/c binding protein | -0.5 |
| 13 | EJK71517.1 | Fucoxanthin-chlorophyll a-c binding protein A | -0.5 |
| 15 | EJK55038.1 | Fucoxanthin-chlorophyll a/c binding protein | -0.4 |
|  |  |  |  |
| **HTHS vs LTHS** | | | |
| 1 | EED93059.1 | Fucoxanthin-chlorophyll a-c binding protein A | +1.3 |
| 2 | XP_002294116.1 | Fucoxanthin-chlorophyll a-c binding protein F | +1.0 |
| 3 | EJK73893.1 | Fucoxanthin-chlorophyll a-c binding protein F | +0.9 |
| 4 | AAB70105.1 | fucoxanthin-chlorophyll a/c light-harvesting protein | +0.5 |
| 5 | EED87666.1 | Fucoxanthin-chlorophyll a-c binding protein | +0.5 |
| 6 | EED95211.1 | fucoxanthin chlorophyll a/c protein | +0.5 |
| 7 | EJK45846.1 | fucoxanthin chlorophyll a/c protein | +0.3 |
| 8 | EED87487.1 | fucoxanthin chlorophyll a/c protein | -0.5 |
| 9 | XP_002288023.1 | fucoxanthin chlorophyll a/c protein | -0.4 |
|  |  |  |  |
| **HTHS vs LTLS** | | | |
| 1 | CAI44219.1 | Fucoxanthin chlorophyll a/c binding protein | -1.1 |
| 2 | EJK56644.1 | Fucoxanthin-chlorophyll a-c binding protein | -1.1 |
|  |  |  |  |
| **HTLS vs LTHS** | | | |
| 1 | XP_002294116.1 | Fucoxanthin-chlorophyll a-c binding protein F | +2.0 |
| 2 | EED93059.1 | Fucoxanthin-chlorophyll a-c binding protein A | +2.0 |
| 3 | EJK73893.1 | Fucoxanthin-chlorophyll a-c binding protein F | +2.0 |
| 4 | XP_002297311.1 | Photosystem I light harvesting protein | +2.0 |
| 5 | EJK46744.1 | Chlorophyll a-b binding protein 1 | +1.7 |
| 6 | CAI44219.1 | fucoxanthin chlorophyll a/c binding protein | +1.7 |
| 7 | AAB70098.1 | fucoxanthin-chlorophyll a/c light-harvesting protein | +1.6 |
| 8 | EED95211.1 | fucoxanthin chlorophyll a/c protein | +1.4 |
| 9 | AAB70106.1 | fucoxanthin-chlorophyll a/c light-harvesting protein | +1.3 |
| 10 | EED87666.1 | Fucoxanthin-chlorophyll a-c binding protein | +1.2 |
| 11 | CAA04226.1 | Fucoxanthin chlorophyll a /c protein | +1.1 |
| 12 | EJK47965.1 | Fucoxanthin-chlorophyll a-c binding protein | +1.1 |
| 13 | AAB70105.1 | fucoxanthin-chlorophyll a/c light-harvesting protein | +0.8 |
| 14 | EJK45846.1 | fucoxanthin chlorophyll a/c protein | +0.8 |
| 15 | EJK58980.1 | Fucoxanthin-chlorophyll a-c binding protein E | +0.7 |
| 16 | CAA04403.1 | fucoxanthin chlorophyll a/c binding protein | +0.7 |
| 17 | EJK55038.1 | fucoxanthin chlorophyll a/c binding protein | +0.6 |
| 18 | EJK71517.1 | Fucoxanthin-chlorophyll a-c binding protein A | +0.5 |
| 19 | EJK56644.1 | Fucoxanthin-chlorophyll a-c binding protein | +0.4 |
|  |  |  |  |
| **HTLS vs LTLS** | | | |
| 1 | XP_002294116.1 | Fucoxanthin-chlorophyll a-c binding protein F | +1.7 |
| 2 | XP_002297311.1 | photosystem I light harvesting protein | +1.1 |
| 3 | AAB70106.1 | fucoxanthin-chlorophyll a/c light-harvesting protein | +0.9 |
| 4 | AAB70105.1 | fucoxanthin-chlorophyll a/c light-harvesting protein | +0.5 |
| 5 | EJK55038.1 | fucoxanthin chlorophyll a/c binding protein | +0.5 |
| 6 | XP_002292353.1 | fucoxanthin chl a/c light-harvesting protein | +0.4 |
|  |  |  |  |
| **LTHS vs LTLS** | | | |
| 1 | EJK47965.1 | Fucoxanthin-chlorophyll a-c binding protein | -1.2 |
| 2 | CAI44219.1 | fucoxanthin chlorophyll a/c binding protein | -1.1 |
| 3 | EJK56644.1 | Fucoxanthin-chlorophyll a-c binding protein | -1.1 |
|  |  |  |  |
